# Effect of Different Network Topologies on Swelling and Mechanical Properties of Polyelectrolyte Hydrogels

**DOI:** 10.1021/acs.macromol.5c03180

**Published:** 2026-02-04

**Authors:** Somesh Kurahatti, Mariano E. Brito, David Beyer, Christian Holm

**Affiliations:** Institute for Computational Physics, 9149 University of Stuttgart, D-70569 Stuttgart, Germany

## Abstract

Elastic modulus, *G*, and equilibrium swelling ratio, *Q*_V_, are two properties of hydrogels, which are linked by the scaling law *G* ∼ *Q*_V_^β^, where β = −1 and −9/4 in the low- and high-salt limits, respectively. Tuning them independently would enable the optimization of the material design for a wide variety of distinct applications. In this work, we investigate several possibilities to achieve this using various network heterogeneities. We employ implicit solvent coarse-grained molecular dynamics simulations to explore mechanical, structural, and thermodynamic properties of hydrogels with varying topologies in comparison to a regular reference gel. We explore regular gels with tetrafunctional cross-linkers arranged in a diamond-lattice fashion, which we take as a reference gel, together with bottlebrush gels, gels with dangling ends, and gels coexisting with floating chains. We observe that incorporating dangling ends changes the swelling ratio and bulk modulus following the relation obtained from the regular reference gel, whereas the bottlebrush and floating-chain gels show stronger deviations. Specifically, floating-chain gels resulted in higher moduli and higher swelling ratios, while bottlebrush gels resulted in lower moduli and lower swelling ratios than the regular counterparts. Concomitantly, a clear change in salt partitioning was observed for various hydrogel architectures. Our results show new ways to optimize the elastic modulus of gels with respect to their swelling behavior and allow for the optimization and on-demand design of hydrogels.

## Graphical abstract

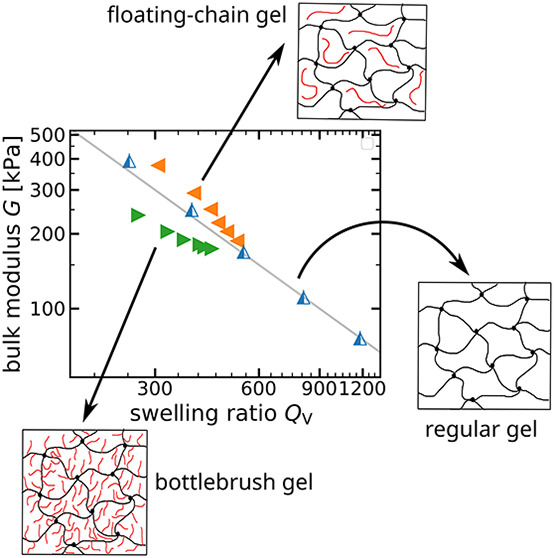

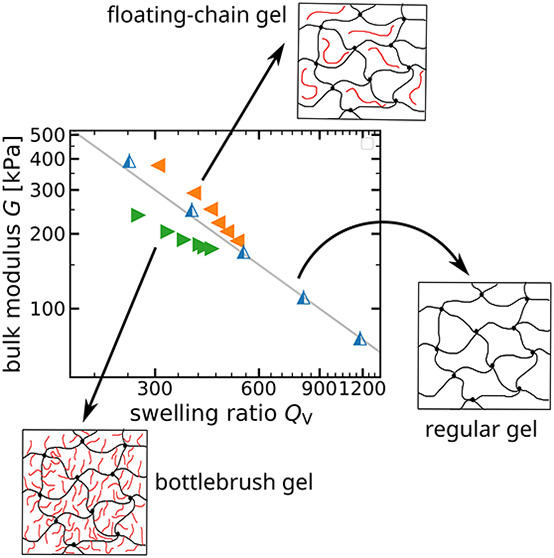

## 1 Introduction

Hydrogels are cross-linked polymer networks that are capable of tremendous swelling when immersed in an aqueous solution, absorbing up to several hundred times their dry mass in water. Ionic hydrogels are composed of polyelectrolyte chains that release ions when dissolved in polar solvents. To maintain electroneutrality, these counterions remain trapped within the gel, generating an additional osmotic pressure that causes the network to swell further.^1^ In addition, the electrostatic repulsion between the charged monomers along the chains contributes both to swelling and to the mechanical integrity of the gel.^2^ Therefore, ionic hydrogels have a swelling capacity much greater than that of their neutral counterparts. A polyelectrolyte hydrogel reaches equilibrium swelling when the counterion osmotic pressure and the electrostatic repulsion of the chains are balanced by the elasticity of the network. When the hydrogel is coupled to a reservoir at a given salt concentration, the ionization of the gel network determines the Donnan potential between the hydrogel and the solution phase in the reservoir, which in turn determines the partition of salt ions between these two phases.^3^

Polyelectrolyte hydrogels are valuable for a wide range of applications in everyday products and advanced biomedical devices,^4–6^ including baby diapers, sanitary products, superabsorbent materials, targeted drug delivery systems, and actuators in microfluidic devices.^7^ In drug delivery systems, their thermodynamic responsiveness is harnessed for controlled drug loading and release. Another interesting application is the use of hydrogels as desalination agents, where the Donnan potential created by the electrolyte networks is used as the driving mechanism in a membrane-free forward osmosis. The solvent confined in the gel phase, which has a lower salinity, is recovered when an external pressure is applied to the gel. To achieve a high desalination efficiency, hydrogels need to simultaneously have a high charge density for enhanced salt rejection and a low modulus for easy compression. The intimate connection among the different hydrogel features makes gels nontrivial materials, which present great challenges when optimizing their design. Different theoretical models for describing swelling have established connections between gel elasticity and osmotic effects, as well as between repulsive chain forces.^8–11^

Scaling theory predicts a simple relation that couples the elastic modulus and the equilibrium swelling ratio of a cross-linked polyelectrolyte gel. This coupling leads to theoretical limitations in hydrogel performance, for example, in the context of desalination efficiency or the development of superabsorbent hydrogels. Determining new gel features that allow us to treat the elastic modulus and swelling ratio in a decoupled manner is crucial. This would allow, for instance, increasing the swelling ability without compromising on the mechanical strength of a gel. Therefore, it is of paramount importance to understand and establish the relation between the elastic modulus and the swelling ratio and in turn link them to the microscopic characteristics of the network.

In a recent study by Arens et al.,^12^ combining experiments and computer simulations, the influence of network architecture on salt partitioning and desalination efficiency was investigated. To this end, hydrogels of various architectures with varying degrees of cross-linking were synthesized and compared. The study revealed that changes in the network architecture simultaneously influence the charge density and swelling capacity. A higher charge density in the swollen gel led to greater rejection of salt ions, while variations in charge distribution due to different network structures had no significant impact. The charge distribution within the hydrogel had no significant effect on desalination efficiency because of the interplay between charge density and mechanical moduli. The weak influence of the architecture on the gel properties arises from the almost invariant topology in the connectivity of the network: the network architecture is varied by altering the network chains, which at the same time determine the elasticity of the network, leaving the type of cross-linking intact.^12^

Alternative network designs, where charged nonelastic dangling ends are introduced and the network cross-linking is altered, are possible as means to achieve networks with different coupling between swelling and mechanical properties.^12,13^ Here, the topological defects across the network, such as dangling ends, grafting, and cross-linking valence, would play a key role in determining the network architecture.^14^ However, despite several experimental studies that support this idea, a systematic theoretical analysis that helps in understanding the network topology is yet to be developed.

Computer simulations are a valuable tool for studying polyelectrolyte hydrogels with a precisely defined network structure, allowing the controlled introduction of various defects. In spite of the experimental work mentioned above, relatively few simulation studies have addressed the role of topological defects in ionic gels. In particular, Edgecomb and Linse investigated the effects of chain length polydispersity and dangling ends formed due to incomplete cross-linking and found that the former decreased the equilibrium gel volume while the latter increased it.^15,16^ In ref 9, chain length polydispersity was taken into account and its effect was analyzed by means of a mean-field Poisson–Boltzmann model, but such theories neglect correlation effects and are based on the simplifying assumption of an affinely deforming network. Furthermore, a scaling theory for complex hydrogel architectures such as bottlebrush macromolecules has been extensively developed in close exchange with simulations^17–20^ providing insight into their swelling behavior and deformation-dependent elastic properties. However, no simulation studies have investigated the mechanical and salt partitioning properties of these complex architectures.

In this paper, we use computer simulations to systematically explore polyelectrolyte hydrogels of various architectures, with the aim of understanding the relationship between network topology and their mechanical and swelling behavior. The article is structured as follows. First, we briefly review the scaling predictions that relate the elastic modulus and equilibrium swelling of polyelectrolyte gels under salt-free and high-salt conditions.^21–23^ In the next step, we introduce our coarse-grained simulation model and compare the scaling predictions against simulations of regular gel networks. Following that, we consider various modifications to the gel network, including dangling ends, bottlebrushes, or floating chains, and we explore the relation between elastic modulus and equilibrium swelling. Finally, we examine the salt partitioning properties of all of the previously explored architectures and discuss the implications of our findings for desalination applications.

## 2 Scaling Theory

We briefly review the scaling theory for the swelling equilibrium of polyelectrolyte hydrogels originally introduced by Dobrynin, Colby and Rubinstein,^23^ with the aim to establish relations between the swelling ratio of the hydrogel and the bulk modulus. This description is based on the theory of semidilute polyelectrolyte solutions because it is assumed that cross-linking does not notably affect the chain conformations. The scaling laws are derived using the concept of blobs, where it is assumed that the polymer chains arrange forming blobs on different characteristic length scales, whose sizes are determined by the polymer entropy and the various interactions present.^24–26^ Assuming that the size of the correlation blob, ξ, is proportional to the persistence length of the chain, it has been observed that for distances larger than ξ, the polymer chain behaves as a (self-avoiding) random walk of the correlation blobs. In contrast, inside a correlation blob, the scaling behavior follows that of a single chain in a dilute solution.^23^

Assuming that the persistence length of the chain is proportional to the electrostatic screening length,^24–26^ the correlation length can be expressed as

1 $$\xi \approx {\left(\frac{B}{\mathrm{cb}}\right)}^{1/2}{\left(1+\frac{2Ac_{s}}{c}\right)}^{1/4}={\left(2Ac_{s}\right)}^{1/4}{\left(\frac{B}{b}\right)}^{1/2}c^{-3/4}+O\left({\left(c/c_{s}\right)}^{1/4}\right)$$

where the parameter *B* depends on the quality of the solvent and counterion condensation, *b* is the size of the monomer, *A* is the number of monomers between effective charges, *c*_s_ is the salt concentration, and *c* is the monomer concentration. In the last equality, we have expanded the expression to the leading term in *c* around *c* = 0. Here, it is additionally assumed that ξ follows an empirical power law dependence of monomer concentration, where the exponent is determined from the condition that the correlation length does not depend on the degree of polymerization *N*.^23^

The osmotic pressure of the swollen hydrogel is given by the sum of both the contribution of the neutral polymer chain Π_p_ and the ionic contribution Π_i_

2 $$\Pi =\Pi_{p}+\Pi_{i}$$

The contribution of the polymer to the osmotic pressure, Π_p_, arises from the configurational entropy^22^ and is given by the concentration of the correlation blobs, which is equivalent to the thermal energy *k*_B_ *T* per correlation volume under semidilute conditions^27^

3 $$\Pi_{p}\approx \frac{k_{B}T}{\xi^{3}}$$

The contribution of ionic osmotic pressure, Π_i_, is the result of the translational entropy of free ions in the gel, which can be expressed as^23^

4 $$\Pi_{i}\approx \left(\frac{c^{2}}{4A^{2}c_{s}+\mathrm{Ac}}\right)k_{B}T$$

which reduces to

5 $$\Pi_{i}\approx \left(\frac{c}{A}\right)k_{B}T$$

in the low-salt limit.^22^

In the free swelling equilibrium, the osmotic pressure due to the polymer chains and counterions is balanced by the elasticity of the network, *G* ≈ Π, where *G* is the isotropic bulk modulus

6 $$G=-V{\frac{dP}{dV}|}_{\mathrm{eq}}$$

at equilibrium conditions, with *P* the total hydrogel pressure and *V* the hydrogel volume. We use this equilibrium condition to establish a relation between *G* and the volume-based swelling ratio

7 $$Q_{V}\equiv \frac{V}{V_{\mathrm{dry}}}$$

where *V* is the equilibrium hydrogel volume and *V*_dry_ is the dry hydrogel volume. The latter can be expressed as

8 $$Q_{V}\approx \frac{1}{b^{3}c}$$

in terms of the monomer size and monomer concentration.

For low salt concentrations (*c* ≫ 2*A*c_s_), the polymeric contribution to Π becomes negligible and the ionic contribution simplifies according to eq 5, resulting in

9 $$G\approx \Pi_{i}\approx \left(\frac{c}{A}\right)k_{B}T=\frac{k_{B}T}{b^{3}AQ_{V}}∼Q_{V}^{\beta },\mathrm{for}\;c\gg Ac_{s},\beta =-1$$

where we have used eq 8 in the last equality. We therefore find that in the low-salt limit, *G* ∼ *Q*_V_^–1^.^22^ Conversely, in the high-salt limit (*c* ≪ 4*A*c_s_) and assuming that the polymeric contribution dominates, we obtain^22^

10 $$G\approx \frac{k_{B}T}{\xi^{3}}∼c^{9/4}∼Q_{V}^{\beta },c\ll Ac_{s},\beta =-2.25$$

where we used eqs 3, 1, and 8, respectively. A comparison of eqs 9 and 10 shows that *G* decreases faster with increasing *Q*_V_ in the high-salt limit.

## 3 Simulation Model and Methods

### 3.1 Coarse-Grained Hydrogel Model

We use the same generic bead–spring model based on the Kremer–Grest model^28^ for the polymer network with implicit solvent and explicit ions as in our previous works.^29–31^ A simulation snapshot of this regular polyelectrolyte hydrogel bead–spring model is shown in Figure 1. Overall, the model is chemically nonspecific, allowing us to focus on generic features rather than atomistic details. The regular gel network consists of linear chains with *N* monomer units, connected by tetrafunctional cross-linkers to form a diamond-like topology. Later on, we introduce various modifications to the network architecture, such as dangling ends and bottlebrushes, and investigate how these topologies influence the network structure. A fraction α of the polymer beads is charged, with a valency of *z* = −1, balanced by an equal number of oppositely charged small ions (counterions) within the simulation box. The networks are characterized by *n*_c_ = 16 chains per simulation box, a backbone chain length of *N* = 20, 25, 30, 37, 45 and a degree of ionization of α = 1.0, unless specified, which means that all beads are charged. For electrostatic interactions, we set the Bjerrum length to λ_B_ = 0. 71 nm = 2σ, which corresponds to an aqueous solution at room temperature. In the simulations, electrostatic interactions are calculated using the P^3^M algorithm,^32^ tuned to a relative accuracy of 10^–4^.^33,34^ Excluded volume interactions are modeled using a Weeks–Chandler–Anderson (WCA) potential^35^ between all pairs of particles

11 $$U_{\mathrm{WCA}}\left(r\right)=\{\begin{array}{ll} 4\epsilon \left({\left(\frac{\sigma }{r}\right)}^{12}-{\left(\frac{\sigma }{r}\right)}^{6}\right) & \mathrm{for}\;r\leq 2^{1/6}\sigma \\ 0 & \mathrm{for}\;r\geq 2^{1/6}\sigma \end{array}$$

where ϵ = *k*_B_ *T* defines the energy scale of the interaction and σ = 0.35 nm corresponds to an effective particle diameter. To model spring-like bonds between polymer beads, we employ the finite-extensible nonlinear elastic potential (FENE)^28^

12 $$U_{\mathrm{FENE}}\left(r\right)=\{\begin{array}{ll} -\frac{1}{2}k_{F}r_{F}^{2}\;\ln\left(1-{\left(\frac{r}{r_{F}}\right)}^{2}\right) & \mathrm{for}\;r<r_{F}\\ \infty & \mathrm{for}\;r\geq r_{F} \end{array}$$

with a spring constant of *k*_F_ = 10 ϵ/σ^2^ and a maximum bond extension of *r*_F_ = 3. 0 σ.

**Figure 1:**
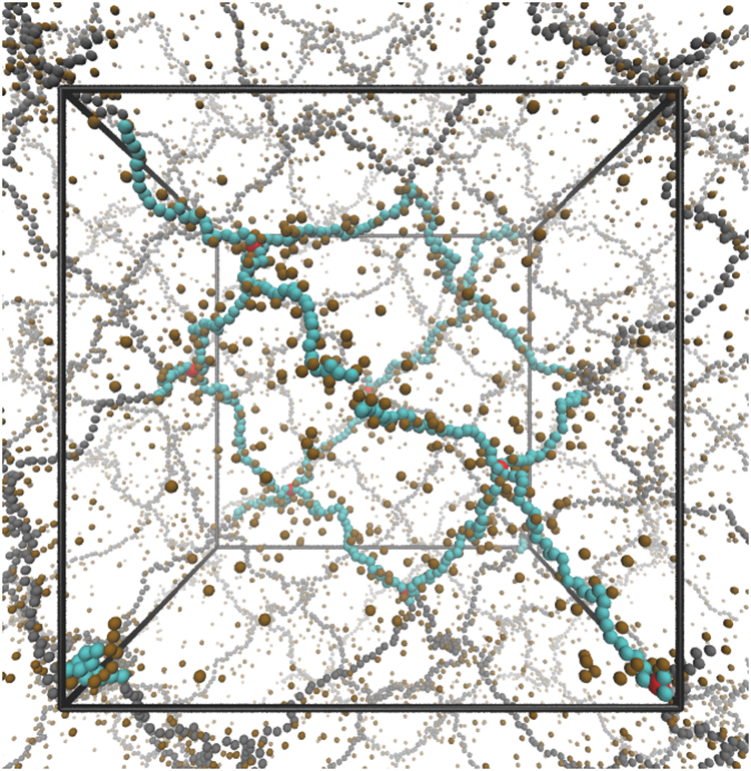
Simulation snapshot of a regular polyelectrolyte hydrogel without any topological defects. Monomers are shown in cyan, cross-linkers in red, counterion in ochre, and all the periodic images in gray.

### 3.2 Investigated Hydrogel Architectures

We explore various hydrogel architectures obtained as systematic modifications of the reference regular diamond-lattice gel network (Figure 1). The purpose of introducing these architectures is to investigate how connectivity defects and charge inhomogeneities modify swelling, elasticity, and salt partitioning relative to the reference regular gel. The schematic illustration of these hydrogel architectures along with their names is shown in Figure 2. The schematic illustrates that a singly detached-chain gel is obtained by cutting one end of the chain that connects to the cross-linker, whereas a fully detached-chain gel is obtained by cutting both ends. A bottlebrush gel is obtained by grafting short side chains onto the backbone network chains, whereas a floating-chain gel is obtained by introducing additional free chains into the regular gel. Compared to the regular gel, these variants possess additional architectural parameters that can be systematically varied to probe a specific physical mechanism.

**Figure 2:**
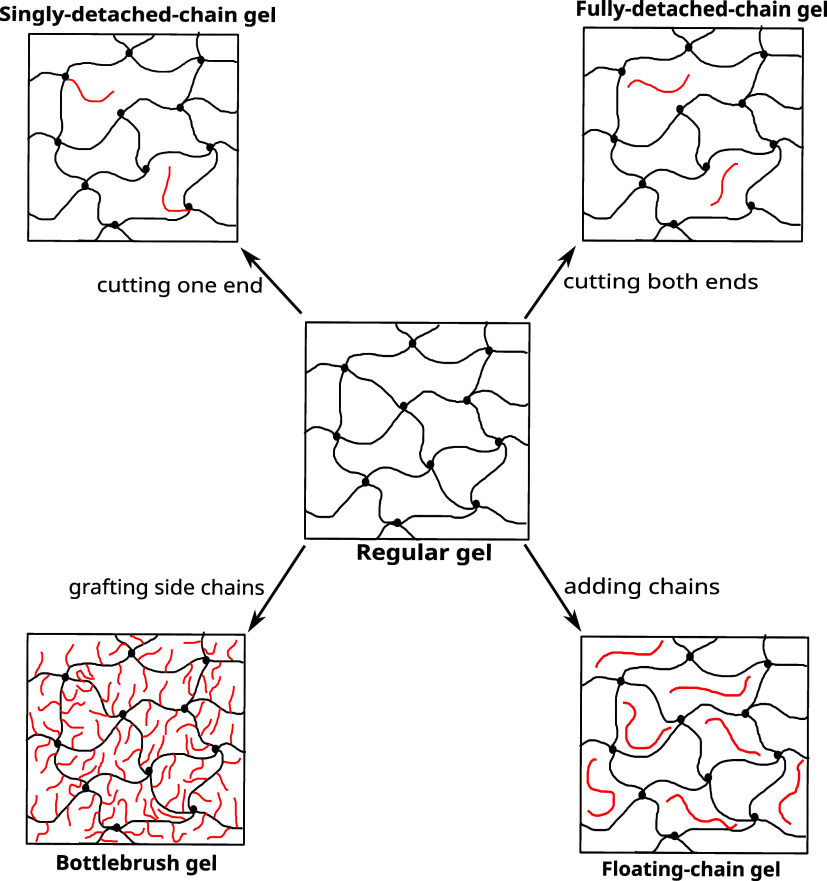
Schematic overview of the investigated hydrogel architectures and their names.

Parameters such as the length of the backbone chain *N*, the charge fraction α, and the salt concentration of the reservoir *c*_s_ are common to all architectures, while other parameters are inherent to specific topologies. In singly detached and fully detached-chain gels, the parameter *n*_DC_ denotes the number of detached strands in the diamond-lattice network. The bottlebrush architecture is characterized by the grafting spacing *m*, namely, the *m*th backbone bead carries a side chain and the degree of polymerization of the side chain *n*. In the floating-chain gel, additional free chains have the same degree of polymerization *N*, as the backbone chains, and their number is denoted by *N*_f_. The parameter values explored for each architecture are given in Table S1.^39^

### 3.3 Simulation Methodology

We use the open-source simulation package ESPResSo^36^ version 4.2.1 to perform the simulations. In the free swelling equilibrium, the system is in thermal, electrochemical, and mechanical equilibrium with the reservoir representing the external salt solution. To ensure the thermal equilibrium, we use Langevin dynamics to sample various conformational states of the hydrogel at a given temperature. For the numerical integration of the Langevin equation, we use the Velocity-Verlet algorithm with a time step of Δ*t* = 0.01 in Lennard-Jones units.^37^ To guaranty the electrochemical equilibrium between the system and the reservoir, we use the grand-canonical Monte Carlo (GCMC) method for the insertion and deletion moves of salt ion pairs according to the specified chemical potential and ensuring electroneutrality of the system.^3,37^ Only ion pairs rather than individual ions are deleted from or added to the simulation box to conserve the electroneutrality, resulting in an electrochemical equilibrium with the reservoir. The excess chemical potential μ^ex^, which enters the acceptance criterion, and the osmotic pressure of the reservoir are determined beforehand from a series of reservoir simulations employing the Widom insertion method.^38^

Lastly, to ensure that the system is in mechanical equilibrium with the reservoir, the total pressure in the system and the reservoir have to be equal. To do this, we perform hydrogel simulations in different box volumes and measured the total virial pressure averaged over time *P*. As a result, we obtain the osmotic pressure of the system, Π = *P* – *P*_res_, as a function of the size of the box (*P*–*V* curve), where *P*_res_, the total pressure of the reservoir, is determined from an independent simulation of the reservoir. The size of the box at which Π vanishes corresponds to the equilibrium volume for the given reservoir salt concentration. To determine this value, we interpolate the simulation data with the phenomenological function *f*(*x*) = *a* + *b*/tan­(*x*–*c*),^31^ as shown in Figure S1.^39^ We calculate the volume-based swelling ratio, *Q*_V_, following eq 7, where the dry volume of the gel, *V*_dry_, is calculated assuming a randomly close packing of WCA beads, ϕ_rcp_ = 0.64, resulting in *V*_dry_ = π*N*_total_σ^3^/(6ϕ_rcp_). Here, *N*_total_ includes all the particles in the simulation box, i.e., monomers and counterions. The bulk modulus *G* in the free swelling equilibrium is calculated by taking the product of the equilibrium volume and the slope of the fitted *P*–*V* curve at the zero-crossing, according to eq 6.

## 4 Results

### 4.1 Validation of Scaling Predictions

We begin our investigation with a validation of the scaling predictions given in eqs 9 and 10, relating *G* and *Q*_V_. To this end, we perform simulations of regular gel networks at various backbone chain lengths *N* and reservoir salt concentrations *c*_s_ for a fixed degree of ionization α = 0.25. Varying the backbone chain length effectively allows us to model hydrogels with different cross-linking densities. In Figure 3a, we plot the measured equilibrium bulk modulus *G* as a function of the swelling ratio *Q*_V_ for different *N* and *c*_s_. The exponents of −1 in the salt-free limit and −2.25 in the high-salt regime, as predicted by the scaling theory, are represented by gray solid lines in the log–log plot. To compare theory and simulations, we fit the data for each salt concentration using an error-weighted linear regression to determine the scaling exponents from the simulations. In the salt-free case, where the osmotic pressure is dominated primarily by counterions, the simulations show agreement with the scaling theory, producing a scaling exponent β = −1.09 ± 0.01. As the concentration of reservoir salt increases, the counterion contribution becomes negligible and the polymeric contribution to the osmotic pressure becomes more important. Concomitantly, the exponent obtained from the simulations decreases, as reported in previous works.^22,40^ For the increasing reservoir salt concentrations of 0.01, 0.05, 0.1, 0.2, and 0.5 M, the corresponding values of β are −1.37 ± 0.04, −2.13 ± 0.07, −2.4 ± 0.1, −2.7 ± 0.2, and −2.6 ± 0.4, respectively. Surprisingly, already at a concentration of 0.05 M, we are close to the predicted value of β = −2.25. For the highest salt concentration considered here (*c*_s_ = 0.5 M), we obtain an exponent of β = −2.6 ± 0.4, which is in agreement with the theoretical predictions within the large error bars. In all cases, we observe that increasing the length of the backbone chain *N* leads to a decrease in the bulk modulus. This behavior is expected because a reduction of the cross-linking density should result in overall softer gels. In Section S2,^39^ we show the plot for the case α = 1.0 with similar scaling exponents in the same range of reservoir salt concentrations.

**Figure 3:**
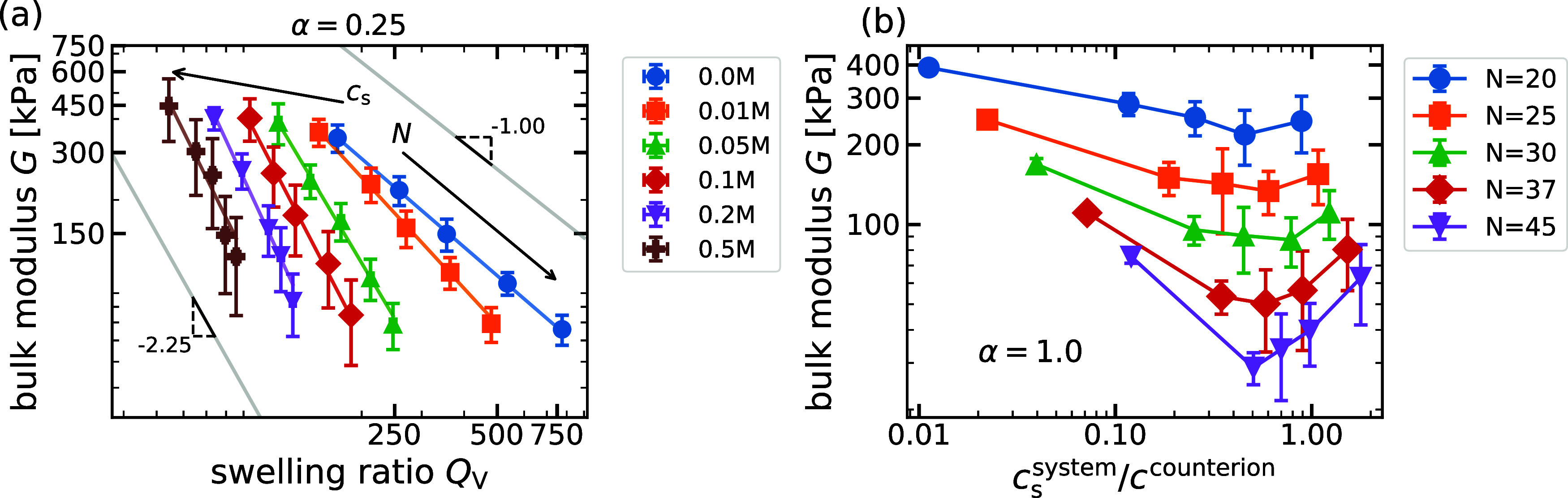
(a) Bulk modulus, *G*, versus the swelling ratio, *Q*_V_, at various salt concentrations, *c*_s_, and backbone chain lengths, *N*. The gray lines with slopes of −1 and −2.25 represent the scaling predictions for low- and high-salt conditions, from eq 9 and 10, respectively. For each *c*_s_, gels with *N* = 20, 25, 30, 37, and 45 are considered. Solid lines of each of the data sets at a fixed *c*_s_ correspond to error-weighted linear regressions. (b) Bulk modulus, *G*, versus system salt concentration normalized by counterion concentration, *c*_s_^system^/*c* ^counterion^, for varying backbone chain lengths *N* = 20, 25, 30, 37, 45 and charge fraction α = 1.0.

To investigate in more detail the salt dependence of the bulk modulus, we plot *G* versus *c*_s_ for varying backbone chain lengths *N* = 20, 25, 30, 37, 45 and the charge fractions of α = 0.25, 1.0 (details can be found in Section S2 ^39^). For α = 0.25, the bulk modulus is shown to increase with *c*_s_ for all values of *N*. For α = 1.0, the bulk modulus exhibits a nonmonotonous trend: initially, it decreases with increasing salt concentration, up to *c*_s_ ≈ 0. 05 M, after which it begins to increase with a further increase in the reservoir salt concentration. The salt concentration at which the bulk modulus begins to increase shifts to higher values with decreasing backbone chain length *N*. This indicates that as the cross-linking density in the hydrogel decreases, the salt concentration at which the elastic modulus starts to rise shifts to lower values. In Figure 3b, we plot the elastic modulus versus the salt concentration of the system normalized by the counterion concentration and observe that the minimums for the chain lengths *N* = 20, 25, 30, 37, 45 occur around *c*_s_^system^/*c* ^counterion^ ≈ 0.5. This plot rationalizes the observed nonmonotonicities for various lengths of network chains *N* by separating the swelling into two regimes, that is, a counterion and a salt-dominated regime. For lower values of *N* = 20, 25, counterions dominate until the reservoir salt concentration reaches 0.2 M. On the other hand, for the chain length *N* = 45, the system is dominated by salt for all *c*_s_ > 0.05 M. The bulk modulus is expected to increase in the salt-dominated regime, i.e., once the salt-to-counterion ratio reaches 1.0, but we observe that it already begins to increase when this ratio is around 0.5. This reduction by a factor of 2 is attributed to the fully charged case (α = 1.0): with *l*_charge_ = σ representing the spacing between adjacent charged monomers, the Manning parameter^41^ Γ = λ_B_/*l*_charge_ = 2 implies that roughly half of the counterions condense onto the network chain, so only the other half remain as free counterions, requiring half the salt concentration to reach the crossover. Hence, inside the gel, the effective salt-to-counterion ratio at the crossover is still 1.

Notably, this nonmonotonicity has not been observed in previous experiments, only an increasing trend is reported independently of the charge fraction.^40,42^ An increasing bulk modulus for a salt-dominated regime is intuitive because higher values *c*_s_ result in higher equilibrium polymer concentrations, leading to an increased polymeric contribution to the osmotic pressure. In contrast, an increase in the elastic modulus for a counterion-dominated regime with decreasing *c*_s_ can be attributed to electrostatic repulsion between network chains in highly charged hydrogels, which causes the network chains to stretch. This stretching leads to more delocalized counterions, which enhances the ionic contribution to osmotic pressure. According to scaling theory, the increase in osmotic pressure must be balanced by the elastic restoring force of the network to achieve the equilibrium swelling volume, thus accounting for the observed enhancement of *G* in the counterion-dominated regime.

### 4.2 Singly and Fully Detached Chains

Having established an overall good agreement between scaling theory and simulations for regular gel networks, we now proceed to investigate hydrogel architectures with topological defects. We consider singly detached-chain gel, which is simply obtained by removing the bond that connects a network chain to one of the cross-linkers, as shown by the simulation snapshot in Figure 4a. This kind of defect can be seen as a simplified representation of the incomplete cross-linking observed in experiments.^43,44^ In contrast to singly detached-chain gel, fully detached-chain gels are formed by removing both ends of the chain from the respective cross-linkers, as shown by the simulation snapshot in Figure 4b. In general, the percolation threshold imposes a constraint on the maximum number of chains that can be detached from an infinite network without compromising its integrity. For a diamond-like topology, the bond percolation threshold is known to be 0.388,^45^ indicating a limitation in which chain removal cannot exceed 38.8% of the total number of chains within the diamond network. Here, to stay below this threshold, we detach a maximum number of *n*_DC_ = 4 chains, both in the case of singly and fully detached-chain gels.

**Figure 4:**
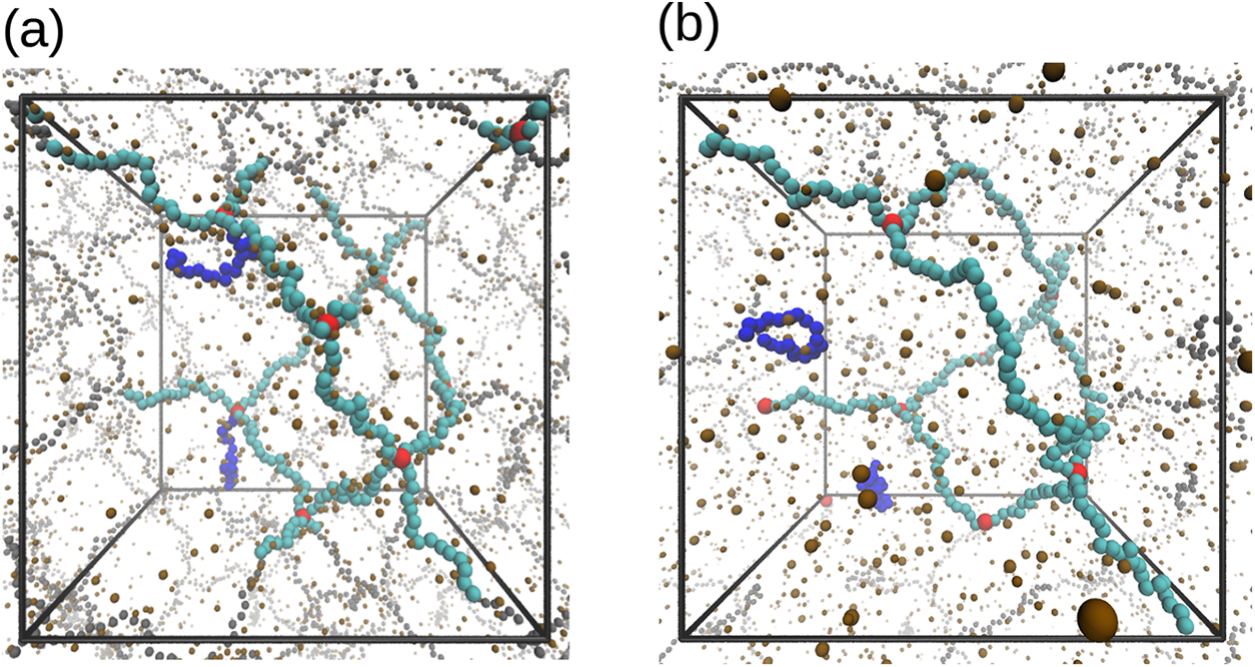
Simulation snapshots of (a) singly detached-chain gel and (b) fully detached-chain gel. Detached chains are colored in blue.

In Figure 5, we plot the bulk modulus *G* versus the swelling ratio *Q*_V_ for singly and fully detached-chain gels. Generally, we observe that an increase in the number of detached chains results in an increase in the swelling capacity of the gel, whereas the bulk modulus decreases. This behavior indicates that with detached chains, there is a trade-off between the swelling ratio and the bulk modulus, analogous to the case of varying the cross-linking density in a regular gel network. Remarkably, the swelling ratio and bulk modulus are coupled in such a fashion that the results are still well-described by scaling theory with roughly the same prefactor. In Figure 5a, for the low-salt regime, exponents β = −1.15 ± 0.03 and −1.05 ± 0.02 are observed for singly and fully detached-chain gels, respectively. For the fits, we used an error-weighted linear regression using all data points for various network chain lengths, *N* = 20, 25, 30, 37, each containing up to 4 detached chains. Similarly, in Figure 5b for a high-salt limit, we observe scaling exponents β = −2.5 ± 0.1 and −2.5 ± 0.2 for singly and fully detached-chain gels. The corresponding data are well-described by the same scaling law with roughly the same prefactor. Although singly and fully detached-chain gels exhibit the same qualitative behavior, we observe that in the low-salt case, fully detached-chain gels exhibit a higher swelling ratio and elastic modulus compared to singly detached-chain gels. In the presence of salt, the large error bars do not allow us to make a definitive statement regarding the modulus. However, fully detached-chain gels do have a higher swelling ratio.

**Figure 5:**
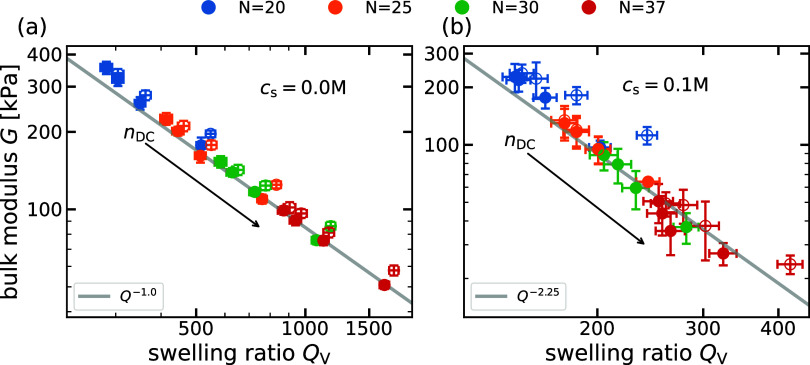
Bulk modulus, *G*, versus the swelling ratio, *Q*_V_, for singly and fully detached-chain gels in the (a) salt-free case and (b) in the presence of *c*_s_ = 0.1 M salt. Gels with four backbone chain lengths *N* = 20, 25, 30, 37 are considered and the number of detached chains, *n*_DC_, is increased progressively from 1 to 4. Error bars are smaller than the symbols in panel (a). The empty and filled markers correspond to singly and fully detached-chain gels, respectively.

To understand why fully detached-chain gels exhibit a higher swelling ratio and bulk modulus in the salt-free limit, we examine the distribution of counterions around the chains, as well as the averaged end-to-end distance *R*_e_ for different types of chains. We determine the number of counterions condensed on various types of chains *N*_cond_, by counting all the counterions within a cutoff distance *R*_c_ = 4σ away from any chain bead. The choice of the cutoff distance is arbitrary; however, we show that our results do not sensitively depend on it in Figure S4.^39^ To make the number of condensed counterions comparable between the different chain types, we normalize it by the total number of counterions and by the total number of chains. Calculating this quantity for regular, diamond-like gels without defects is straightforward as all of the chains are identical. However, for singly and fully detached-chain hydrogels, there are two types of chains: detached chains and intact chains within the network.

An examination of the data in Table 1 reveals that counterion condensation, *N*_cond_, onto the intact chains is lower for a network with fully detached chains (*N*_cond,intact_^fully^ = 0.0433) than in the case with singly detached chains (*N*_cond,intact_^singly^ = 0.0473) for *n*_DC_ = 4. This is related to an end-effect, since for finite chains the electrostatic field is lower for a stretched chain.^46,47^ Furthermore, the difference *N*_cond, intact_^singly^ – *N*_cond,intact_^fully^ depends on the number of detached chains *n*_DC_ and becomes more pronounced as the number of detached chains increases, as shown in Figure 6. Other values of *n*_DC_ are shown in Table S2.^39^ The reduced counterion condensation on the network for fully detached-chain gels can be attributed to free chains floating in the simulation box, which delocalize the counterions and increase their entropy. This leads to an increase in swelling and to a more homogeneous distribution of ions. Thus, the screening near the backbone chain decreases, resulting in increased intrachain repulsion when detached chains are present. Consequently, the end-to-end distance *R*_e_ of intact chains is higher in the case of fully detached chains compared to the case of singly detached chains, and this difference increases with *n*_DC_.^39^ As demonstrated in Figure 6, fully detached-chain gels lead to reduced counterion condensation in the network, leading to higher osmotic pressure due to ions. According to scaling theory, the elasticity of the chain network, *G*, must balance the total osmotic pressure, Π = Π_p_ + Π_i_, resulting in a higher elastic modulus for fully detached-chain gels, as demonstrated in Figure 5a.

**Table 1:** Number, *N*_cond_, of Condensed Counterions per Chain and Averaged End-to-End Distance, *R*_e_, for Detached and Intact Chains in Gels

*n*_DC_ = 4		*N*_cond_	*R*_e_
regular		0.0483	0.744 ± 0.002
singly	detached	0.0285	0.626 ± 0.003
	intact	0.0473	0.775 ± 0.002
fully	detached	0.0327	0.558 ± 0.002
	intact	0.0433	0.787 ± 0.002

**Figure 6:**
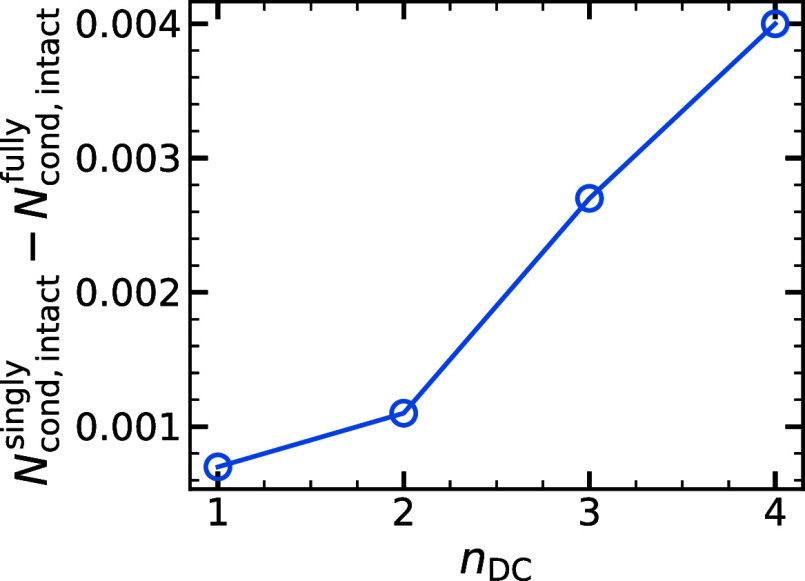
Difference in counterion condensation on the intact chains of the singly and fully detached-chain gels, *N*_cond,intact_^singly^ – *N*_cond,intact_^fully^ versus the number of detached chains, *n*_DC_.

Overall, our analysis reveals that fully detached chains lead to a stretching of the network chains, and consequently, the counterion density is reduced in their vicinity. This offers a mechanism for reducing the screening on the backbone chains, affecting the network swelling and bulk modulus.

### 4.3 Bottlebrushes and Floating Chains

So far, we have found that detached-chain gels follow the same scaling law between the swelling ratio *Q*_*V*_ and the bulk modulus *G* as the regular gel networks. Therefore, topological defects do not offer a clear path to the design of networks with an alternative coupling between *Q*_V_ and *G*. Motivated by the simulation results in the last section and by experimentally synthesized gels,^17–19^ we now explore whether a different coupling or decoupling can be achieved by using bottlebrush gels and floating-chain gels in the salt-free case. In a bottlebrush gel, additional length *n* polymer strands are grafted to the network backbone chains at each *m*th monomer, as shown in the schematic of Figure 7a, leading to a gel with *N*_tot_ = 16*N* (1 + *n/m*) monomers. For the simulation study, we consider *n* = 4 and *m* = 2, 4, 6, 8, 10, 12. A representative example of a bottlebrush gel is shown in the simulation snapshot in Figure 7b for *m* = 4 and *n* = 6.

**Figure 7:**
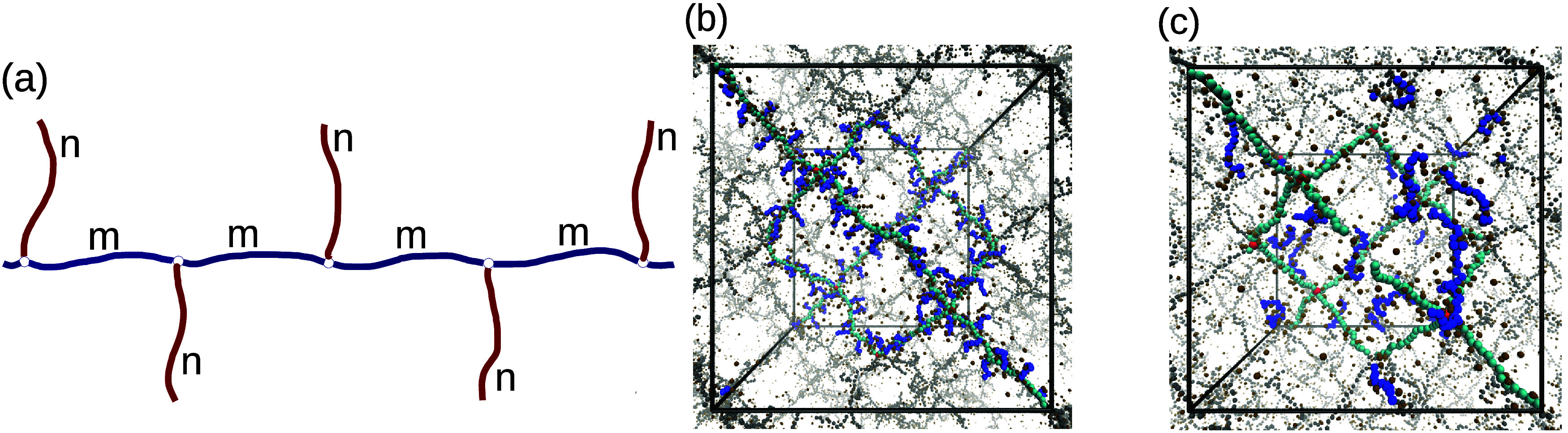
(a) Schematic of the bottlebrush architecture with (*N*, *n*) being the degree of polymerization of the backbone chain and side chains, and *m* being the spacing between side chains. (b) Simulation snapshots of a polyelectrolyte bottlebrush gel (*m* = 4, *n* = 6) and (c) floating-chain gel (*N*_f_ = 16).

Inspired by the results for fully detached-chain gels of the previous section and with the aim of understanding the extent of counterion *delocalization* on the scaling relations, we also consider a floating-chain gel: a regular gel network containing *N*_f_ added free-floating chains. Although experimentally controlling the number of floating chains in the gel can be difficult, this architecture serves as an interesting case for the study as a counterpart to the bottlebrush case. For the study, we take the floating chains to have the same degree of polymerization as the backbone chain, *n* = *N*. We have made this choice, as a different value did not produce fundamentally different results from those shown in Figure S5.^39^ A representative snapshot of this case is shown in Figure 7c for *N* = 20 with *N*_f_ = 16. Finally, we consider the grafted and floating chains as well as the backbone chains to be fully ionized.

Figure 8a,b shows how the equilibrium volume of the gel, *V*_eq_, and the bulk modulus, *G*, change when varying the degree of polymerization of the backbone chains for the introduced architectures and the regular gel. As expected, *V*_eq_ increases with *N* for all three cases, as depicted in Figure 8a. The regular gel follows the theoretical scaling, which is obtained assuming that the gel volume is proportional to the cube of the end-to-end distance of the chains with fully charged chains where *R*_e_ ∝ *N*.^30^ The equilibrium volume then scales as *V*_eq_ ∝ *R*_e_^3^ ∝ *N* ^3^ with the degree of polymerization *N*. Upon fitting the *V*_eq_ for the reference regular gels, we obtain a value close to the theoretically predicted scaling *V* ∼ *N* ^2.90^, as represented by a blue solid line.

**Figure 8:**
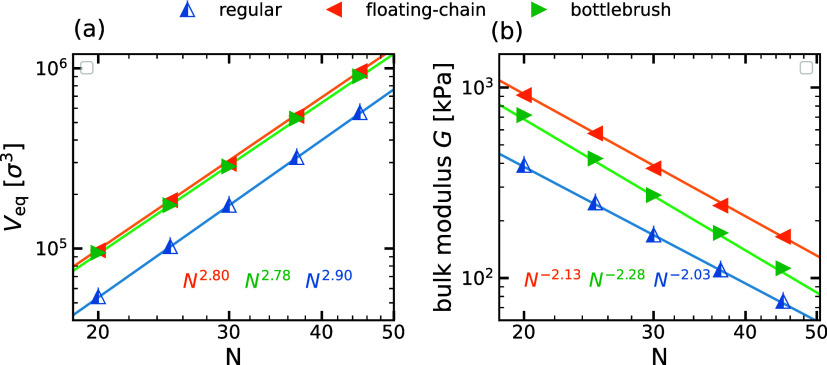
(a) Equilibrium swelling volume, *V*_eq_, and (b) bulk modulus, *G*, versus chain length, *N*, for the bottlebrush gel and floating-chain gel of fixed architectural parameters *m* = 2, *n* = 6, and *N*_f_ = 32, respectively, compared to the reference regular gel of same chain length.

The equilibrium volume of the new gels is larger than that of the reference case because of the increased osmotic pressure resulting from the incorporation of charges with the extra grafted/floating chains. Interestingly, the equilibrium volume of bottlebrush gels and floating-chain gels scales as *V*_eq_ ∼ *N* ^2.80^ and *V*_eq_ ∼ *N* ^2.78^, respectively, since additional effects are contributing. For bottlebrush gels, the strong local charge density introduced by the presence of the grafted chains leads to an increased self-repulsion of the chains together with a perturbation of the condensation layer around the backbone chains. This results in a stronger swelling compared to the regular gel reference case. At higher values of *N*, this stronger swelling becomes less pronounced as the length of the grafted chains becomes much smaller than the length of the backbone chain. Similarly, as pointed out in the last section, the charged floating chains delocalize the condensed counterion on the backbone chains, producing a swelling that is even stronger than that in the bottlebrush case.

Regarding the bulk modulus *G*, we observe that it decreases with increasing *N* for all cases. *G* for the regular gel scales with the theoretically predicted law, *G* ∝ *N* ^–2^,^23,48^ as shown by the solid blue line. We notice that this scaling holds for all strongly cross-linked gels, independent of the charge on the chain and salt concentration.^22^

For the new networks, the higher local charge density leads to a larger *G* for constant *N* compared to the regular case. Our simulations predict a scaling *G* ∝ *N* ^–2.13^ and *G* ∝ *N* ^–2.28^ for floating-chain gels and bottlebrush gels, respectively. This indicates that for large *N*, the modulus of bottlebrush gels is closer to that of regular gels, which is expected as the side chains behave as effective monomers. In contrast, for floating-chain gels, the delocalization of condensed counterions produces an effective repulsion stronger than the repulsion between brushes, resulting in a substantial enhancement of *G*.

In Figure 9, we analyze the scaling relation between *G* and *Q*_V_. Figure 9a shows the scaling of *G* with *Q*_V_ with varying *N* for the systems in Figure 8. We notice that the bottlebrush and floating-chain gels describe approximately the same universal scaling as the reference regular gel and the theoretical prediction in eq 9, for varying cross-linking density, namely, *N*. This observation suggests that changing the density of cross-linking for fixed architectural parameters (*m*, *n* or *N*_f_) allows varying the prefactor γ relating the swelling ratio and the bulk modulus (*G* ∝ *γQ* ^β^) so that different values for *G* can be obtained for the same *Q*_V_ and vice versa.

**Figure 9:**
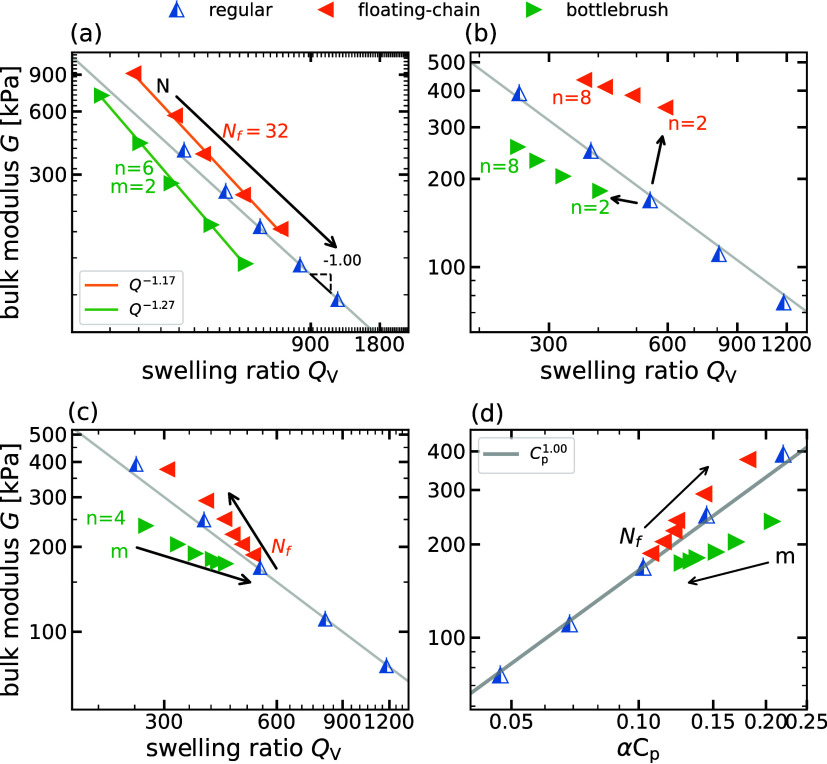
Bulk modulus, *G*, versus the swelling ratio, *Q*_V_, for various hydrogel architectures in the salt-free case. (a) Regular gel, bottlebrush gel (*m* = 2, *n* = 6), and floating-chain gel (*N*_f_ = 32) for different backbone chain lengths *N* = 20, 25, 30, 37, 45. (b) Bonds connecting side chains to the backbone chain are removed to form floating chains, allowing for a direct comparison between bottlebrush gels and floating-chain gels. The spacing between adjacent side chains is set to *m* = 4. Essentially, side chains in the bottlebrush gel become floating chains in the floating-chain gel when the bonds connecting side chains to the network are removed. (c) Bottlebrush gels (*n* = 4) and floating-chain gels for varying numbers of side chains, *m* = 2, 4, 6, 8, 10, 12, and floating chains, *N*_f_ = 2, 4, 6, 8, 16, 32, respectively, are added to the gel with the backbone chain length *N* = 30. (d) Bulk modulus, *G*, versus equilibrium polymer concentration, α*C*_p_, for the same system considered in Figure 9.

This figure shows the relationship between *G* and *Q*_V_ when we vary the length *n* of the grafted chains and the floating chains. For both cases, the relation between *G* and *Q*_V_ scales similarly under the variation of *n*, for constant cross-linking density. This relation is weaker than the universal reference scaling, that is, with a scaling exponent |β| < 1. Interestingly, the figure shows the relevance of the location of the additional chains relative to the backbone structure in determining the gel properties. We notice that floating chains result in a remarkably higher bulk modulus and swelling ratio. This is mainly a result of the counterion delocalization that floating chains produce: the larger entropic contribution of the delocalized counterions contributes to swelling, whereas the reduction of the backbone chain screening also promotes swelling and increases the bulk modulus simultaneously. Figure S6 ^39^ shows the reduced counterion condensation profile around the backbone chains of the floating-chain gels compared to the bottlebrush gels for the parameters (*m*, *n*) considered in Figure 9b.

In the new gels, alternative property modifications can be made by modifying the number of extra chains coexisting with the backbone strands. For bottlebrush gels, we increase the number of extra chains by increasing the grafting density, namely, decreasing *m* while simply increasing *N*_f_ in the floating-chain case. In Figure 9c, we observe that the variation of a grafting density establishes a different relation between *G* and *Q*_V_ for constant *N*, compared to the universal reference. The simulations show a weaker dependence of *G* on *Q*_V_. Variation of grafting density also opens the possibility of creating networks with a smaller swelling and larger bulk modulus than the regular counterpart, without affecting the cross-linker density.

Alternatively, the floating-chain gel leads to a slightly stronger coupling, namely, a scaling exponent |β| > 1, between *G* and *Q*_*V*_ compared to the universal reference in eq 9. At constant cross-linker density, increasing *N*_f_ for floating-chain gels lead to larger *G* values compared to the regular counterpart, with less impact on swelling. For these systems, the counterion condensation profiles for network architectures are shown in Figure S6a.^39^ Similar to the previous case, we observe a remarkable ion delocalization, which is more pronounced at larger *N*_f_.

Finally, in Figure 9d, we show the dependence of the bulk modulus on the equilibrium polymer charge concentration α*C*_p_ for the same parameters as in Figure 9c. The scaling behavior *G* ∼ α*C*_p_ correctly fits the observed dependency for regular gels, while the bottlebrush gels and floating-chain gels deviate from this fit. A different bulk modulus observed at the same polymer concentration reveals different charge distributions within various architectures, namely, floating-chain gels have a more homogeneous distribution of charges, while bottlebrush gels have regions of high local charge density. Comparable observations are made in the presence of salt, and this phenomenon is discussed in greater detail in the following section.

#### 4.3.1 Mechanical Properties in the Presence of Salt

So far, we have established that the introduction of floating chains and the possibility to vary the grafting density in bottlebrush gels allows us to tune the coupling strength between the bulk modulus *G* and the swelling ratio *Q*_V_ in the salt-free case. We now explore whether a similar deviation from the scaling law holds in the presence of salt. We use the same parameters for bottlebrushes, floating chains, and the network with dangling ends, as used in Figure 9c, and the system is coupled to a reservoir with salt concentration *c*_s_ = 0.01 M. In Figure 10a, we observe a similar relationship between *G* and *Q*_V_ for all topological defects investigated and an overall decrease in the swelling ratio due to the presence of salt ions. As explained in Section 4.1, an intermediate scaling exponent β = −1.55 between the low- and high-salt limits of the scaling theory is obtained for regular gels. Despite the presence of salt, gels with dangling ends follow the same scaling behavior as the regular case. A comparison of the *G* versus *Q*_V_ graphs with increasing reservoir salt concentration *c*_s_ = 0.0, 0.01, 0.1 M (see also Section S6 ^39^) reveals that the differences in the swelling ratios and the bulk modulus of the gel with changing architectural parameters (*N*_f_ and *m*) progressively decrease. It is interesting to note that even at a higher salt concentration, bottlebrush gels still clearly distinguish themselves from the reference case. In contrast, the floating-chain case tends to cluster and come closer to the regular gel. This is a consequence of how screening affects the interaction of backbone chains with grafted or floating chains.

**Figure 10:**
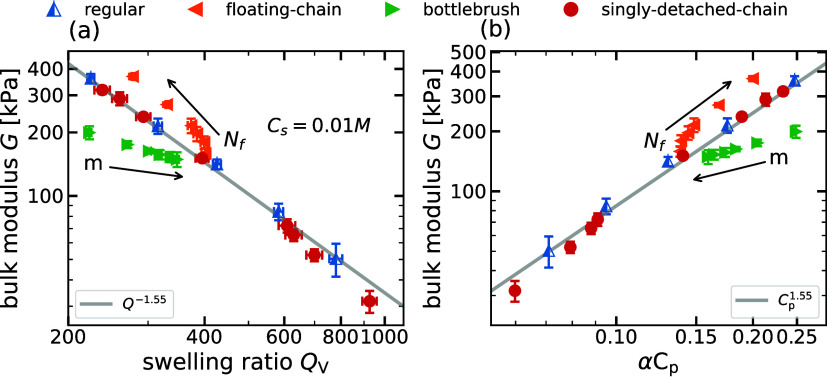
(a) Bulk modulus, *G*, versus the swelling ratio, *Q*_V_, of networks of various architectures at salt concentrations *c*_s_ = 0.01 M. The gray line with slopes of −1.55 represents the error-weighted linear regression fit to the bulk modulus values of regular gels. The network chain lengths for regular gels are *N* = 20, 25, 30, 37, and 45. The floating chains and bottlebrushes are added to the gel with *N* = 30. The number of detached chains *n*_DC_ = 1, 2, 3, 4 are considered for gels with two backbone chain lengths *N* = 20, 37. (b) Bulk modulus, *G*, versus equilibrium polymer concentration, α*C*_p_, for the same system considered in Figure 10.

In Figure 10b, we show the dependence of the bulk modulus on the equilibrium polymer charge concentration α*C*_p_. Similar to the salt-free case in Figure 9, we combine the relation *G* ∼ *Q*_V_^–1.55^ from Figure 10a with eq 8 to derive the scaling law *G* ∼ α*C*_p_^1.55^ (more details can be found in the Supporting Information^39^). This scaling behavior is shown in Figure 10b and accurately captures the trends for both regular gels and singly detached-chain gels.

The different mechanical strengths observed at the same polymer concentration can thus be explained by different charge distributions within various architectures, which in turn leads to different electrostatic microenvironments in the networks. In the next section, we discuss that the excess chemical potential of the ions within the gel depends on this charge distribution, which determines the partitioning of the salt.

### 4.4 Salt Partitioning

As explained in Section 1, the partitioning of salt between a polyelectrolyte gel and an aqueous solution is nonuniform and can be leveraged in desalination applications. The partitioning of salt is typically quantified using the partition coefficient

13 $$\xi \equiv \frac{c_{s}^{g}}{c_{s}}$$

which is the ratio of the salt concentration within the gel, *c*_s_^g^, to the salt concentration in the reservoir, *c*_s_. The Donnan theory yields a simple expression for the partition coefficient in terms of polymer concentration *c*_p_ and the difference Δμ ≡ μ_res_^ex^ – μ_gel_^ex^ between the excess chemical potential of an ion pair in the gel μ_gel_^ex^ and in the reservoir μ_gel_^ex^ ^39^

14 $$\xi =-\frac{\alpha c_{p}}{2c_{s}}+\sqrt{{\left(\frac{\alpha c_{p}}{2c_{s}}\right)}^{2}+\exp\left(\beta \Delta \mu^{\mathrm{ex}}\right)}$$

This equation is completely general, but deceptively simple: Because excess chemical potentials depend on various concentrations and interactions, the equation has to be solved self-consistently and cannot be evaluated in most cases. However, it serves as an important starting point for discussions of the influence of electrostatic interactions on the partitioning behavior.

For the case Δμ^ex^ = 0, the “ideal Donnan theory”, the partition coefficient can be evaluated in a closed form. In Figure 11, we show the salt partition coefficient as a function of the polymer charge concentration α*c*_p_ for a reservoir salt concentration of *c*_s_ = 0. 01 M and different hydrogel architectures. The ideal Donnan theory, shown as a dashed line, predicts the partition coefficient to be a universal function of α*c*_p_/*c*_s_, irrespective of the structural details of the gel. Furthermore, it predicts an increase in the rejection of salt from the gel as the polymer concentration is increased.

**Figure 11:**
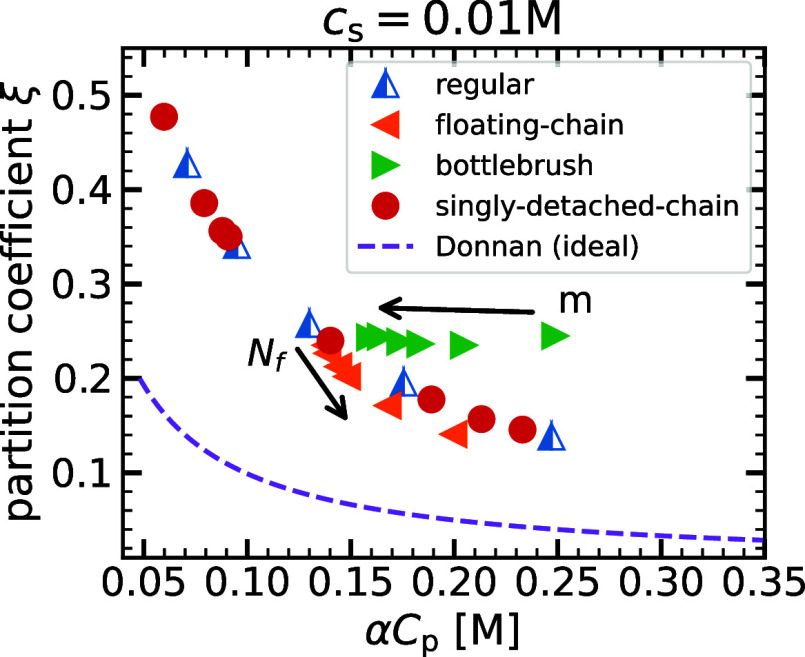
Partition coefficient, ξ, of monovalent salt versus the polymer charge concentration, α*c*_p_, for different network architectures. The network chain lengths for regular gels are *N* = 20, 25, 30, 37, 45. Bottlebrush gels (*n* = 4) with varying numbers of side chains, *m* = 2, 4, 6, 8, 10, 12, and floating-chain gels, with *N*_f_ = 2, 4, 6, 8, 16, 32, are considered for network chain length *N* = 30. For singly detached-chain gels, the values *n*_DC_ = 1, 2, 3, 4 are considered for two backbone chain lengths *N* = 20, 37.

Qualitatively, the simulation data for all considered architectures follow the behavior expected in the ideal Donnan case: As the polymer charge concentration increases, salt gets increasingly rejected from the gel, leading to a lowering of the partition coefficient. However, quantitatively, the partition coefficient is enhanced as compared to the ideal Donnan theory for all cases. This behavior is in agreement with previous works, which found that the partition coefficient in strong polyelectrolyte hydrogels is higher than predicted by the ideal Donnan theory.^30^ The observed discrepancy between the ideal theory and simulation results arises due to electrostatic correlation effects, which are encoded in Δμ^ex^ but neglected in the ideal theory: because the overall charge density inside the system is higher than in the reservoir, the excess free energy cost of inserting an ion pair into the system is negative, i.e., Δ*F* ^ex^ = −*β*Δμ^ex^ < 0.^49^ Thus, the enhancement of the partitioning compared to the Donnan theory prediction is a consequence of the stronger electrostatic interactions inside the gel compared to the reservoir, favoring a stronger uptake of salt in the interacting case. Despite this enhancement, the observed partition coefficients are all smaller than unity, i.e., salt is always rejected from the gel. The same qualitative behavior is also observed at a different salt concentration (Figure S9 ^39^).

In addition to the differences between ideal theory and interacting systems, we can also identify clear distinctions between the salt partitioning for different hydrogel architectures. Because these distinctions occur when the data are plotted as a function of the polyelectrolyte charge concentration, we can conclude that they arise due to differences in the charge distribution within the simulation box. This in turn affects Δμ^ex^ of an ion pair and thus the partitioning behavior.

Comparing the different architectures, we observe that the partition coefficients for regular gels and singly detached-chain gels fall onto the same curve, similar to the mechanical properties in Figure 5. In particular, increasing the number of detached ends *n*_DC_ = 1, 2, 3, 4 leads to a reduced equilibrium polymer concentration and improved partition coefficients in a manner analogous to the behavior observed when decreasing the cross-linker density of reference regular gels. This observation suggests that regular gels and singly detached-chain gels exhibit very similar charge distributions, leading to a similar electrostatic environment for the ions. In contrast, both bottlebrush gels and floating-chain gels deviate from this behavior, again mimicking the mechanical properties. For floating-chain gels, we observe that the partition coefficient is lowered compared to the regular gel case, i.e., the partitioning is closer to the ideal behavior. We can explain this effect by considering that the floating chains are essentially distributed uniformly over the whole simulation box, leading to a more “smeared out” uniform background charge. In general, this charge distribution results in a smaller value of Δμ^ex^ than for a regular gel at the same charge density, which diminishes the energetic enhancement of the partitioning. In contrast, the partition coefficient is the highest for a bottlebrush gel. For the bottlebrush gel, the inhomogeneity of the charge distribution is further enhanced in comparison to that of the regular gel because the grafted side chains lead to localized regions with a very high polymer charge, whereas the regions in between the chains are devoid of charges. The localized regions with high polymer and counterion charge density are very favorable for the insertion of salt ions because of the high charge density. Consequently, Δμ^ex^ and thus the partition coefficient increase strongly for the bottlebrush architecture.

For desalination applications, a strong salt rejection, i.e., a low partition coefficient, is desired. Our simulation results demonstrate that the salt rejection of an ionic hydrogel strongly depends on the local arrangement of polymeric charges and not only the overall charge density within the gel. This insight suggests that the architecture of a hydrogel can be tuned to optimize salt rejection. As an overall guideline, to maximize salt rejection, one should try to synthesize hydrogels with a charge distribution that is as uniform as possible. We are aware that in experiments it can be difficult to synthesize and control the amount of free-floating chains because they would diffuse out when the hydrogel is washed. As a viable alternative, we propose in Figure 12 the insertion of floating chains that are connected to the network chain by a neutral linker chain, allowing them to be evenly distributed throughout the gel. In addition to desalination, there are also complementary use cases of hydrogels, where enhanced salt partitioning is highly desired. This is, for example, the case in sequestration applications, e.g., the use of hydrogels to filter toxic ions out of contaminated water.^50^ In these cases, bottlebrush architectures may be an efficient way to enhance the partitioning of salt into a gel.

**Figure 12:**
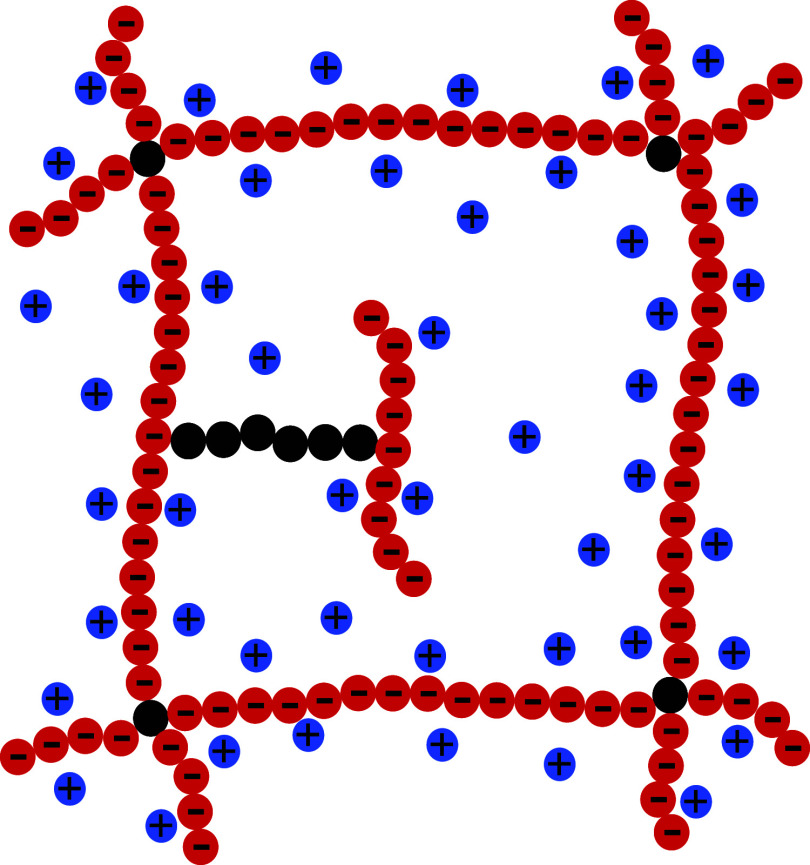
A floating chain held onto the backbone network chain of the gel through a neutral linker chain.

## 5 Conclusion

In this work, we studied the swelling and elastic properties of polyelectrolyte gels with various architectures, in particular, the predicted relationship between the degree of swelling and the bulk elastic modulus. Previous scaling models predicted two distinct power law dependencies between the equilibrium swelling degree and the elastic modulus in the salt-free case and in the high-salt limit for a regular polyelectrolyte gel. With the help of coarse-grained simulations, we have explored the influence of topological defects on these properties and scaling relations. For modeling the polyelectrolyte networks, we have introduced a generic coarse-grained bead–spring model, which allows us to create gels with different architectures and topologies, namely, regular diamond-lattice gel networks, singly and fully detached-chain gels, and bottlebrush and floating-chain gels. For the commonly used regular diamond-lattice gel, we validated the simulation model against theoretical predictions that relate swelling to the bulk modulus, obtaining results in good agreement in the high- and low-salt limits. Moreover, we extended the exploration for intermediate salt concentrations, leading to coherent results. For networks with topological defects, namely, singly and fully detached-chain gels, we found that the incorporation of defects leads to a smaller bulk modulus *G* and larger swelling *Q*_V_, preserving, however, the theoretical scaling predictions between *G* and *Q*_V_ at zero added salt, *G* ∼ *Q*_V_^β^ with β = −1. The increase in swelling and decrease in bulk modulus when defects are added to the (backbone) network reflect the key role of the cross-linkers in distributing the pressure on the chains and the consequent elastic response of the network. Preservation of the scaling law supports the usage of elastic models that neglect cross-linking to a qualitative extent,^10,11,30^ but cross-links are a key contribution in the development of quantitative and accurate descriptions.^11^ We also observed that gels with fully detached ends show an enhanced bulk modulus and swelling ratio compared to singly detached ends, due to the reduced counterion location on the network, induced by fully detached chains. Counterion condensation is a phenomenon widely studied with deep consequences in determining gel properties: condensed ions reduce the osmotic pressure and screen the self-repulsion of the backbone chains, affecting *G* and *Q*_V_. We have shown here that topological defects can be used to manipulate the condensation and alter the condensation layer, thus modifying the gel properties in a desired direction.

To counteract the observed trade-off between the swelling ratio and the bulk modulus, we modified the topology of the backbone chains in the network through the introduction of complex topologies. To this end, we investigated hydrogels with different structures, namely, bottlebrush gels and floating-chain gels. Floating-chain gels present some experimental challenges, as osmotic pressure effects have to be overcome to prevent the chains from leaving the gel, for example, by considering sufficiently long floating chains. Nevertheless, they represent an interesting case study as a counterpart to the bottlebrush case, helping us to understand the topological role of grafting. The addition of the excluded volume and electrostatic repulsion of the additional strands alters the conformation of the backbone chains, modifying the swelling and bulk modulus in a different way from the previous topological defects. Although the new architectures might lead to larger *G* and *Q*_V_ than the corresponding regular diamond-lattice network, the effect of the variation of the cross-linking density in the new architectures does not substantially alter the scaling between *G* and *Q*_V_. However, these networks possess novel architectural parameters that establish alternative connections to *G* and *Q*_V_. We observed that a stronger or weaker *G*–*Q*_V_ coupling can be found by varying the amount of extra chains, freely floating or grafted, respectively. We also observed that variations in the degree of polymerization of the extra chains *n* lead to a weaker *G*–*Q*_V_ coupling, resulting in mixed effects concerning swelling and bulk modulus. For identical parameters, increasing *n* at fixed *m* raises both *G* and *Q*_V_ of floating-chain gels significantly more than in bottlebrush gels. These effects can be explained by the perturbation of the counterion localization around the backbone chains either by dragging them away, in the case of the floating chains, or by overlapping and excluded volume, in the case of the bottlebrush gels.

Finally, we compared the salt partitioning properties of various architectures. We found that gels with an increasing number of dangling ends result in a reduced equilibrium polymer concentration and an enhanced partition coefficient similar to the data points obtained by decreasing the cross-linker density, i.e., hydrogels with dangling ends and varying *N*. This is closely related to the finding that the mechanical properties of networks with dangling ends align with the scaling prediction of regular networks. However, the salt partitioning properties of gels incorporating bottlebrushes and floating chains deviated from the trend observed in regular gels and singly detached-chain gels. This reveals how inhomogeneities in the charge distribution affect the partitioning behavior. These results indicate that the hydrogel architecture can be tuned to optimize salt rejection.

In summary, we have explored the effects of the topology and topological defects on the equilibrium properties of the polyelectrolyte hydrogel. We have shown that manipulation of the topology offers a means of customizing gel properties, potentially enabling greater versatility and tunability in various applications.

## Supplementary Material



## Notes

Additional *P*–*V* curves; plots of *G* vs *Q*_V_, *G* vs *c*_s_, and *G* vs α*C*_p_ and partition coefficients at a higher salt concentration for all the network architectures; and cumulative counterion distribution profiles together with tables and theoretical calculations^39^ (PDF)
