# Supplementary material for: Effect of Different Network Topologies on Swelling and Mechanical Properties of Polyelectrolyte Hydrogels
Source: Macromolecules. 2026 Feb 4;59(4):1918–30. doi: 10.1021/acs.macromol.5c03180 (PMC12947679; doi:10.1021/acs.macromol.5c03180)
Supplement: Supplementary file 1 [file ma5c03180_si_001.pdf]

Supporting Information: Effect of Different Network Topologies on Swelling and Mechanical Properties of Polyelectrolyte Hydrogels

Somesh Kurahatti, Mariano E. Brito,* David Beyer, and Christian Holm

Institute for Computational Physics, University of Stuttgart, D-70569 Stuttgart, Germany

E-mail: mbrito@icp.uni-stuttgart.de

1 Exemplary P-V Curves and Table of Parameters

As explained in the main text, a P-V protocol is employed to determine the equilibrium swelling volume of the hydrogel in the presence of an external salt solution. In Figure S1 we plot the length of the simulation box containing the diamond lattice hydrogel versus the averaged virial pressure recorded inside the hydrogel, which yields a pressure-volume curve (P-V). For the exemplary curves shown in Figure S1, simulations are performed in a semi-grand-canonical ensemble where the gel can exchange salt/counterions with the reservoir. In the salt-free case ($c_\mathrm{s} = 0$), the total pressure inside the gel must balance $P_\mathrm{gel} = 0$. The zero crossings in the PV curve correspond to the swelling equilibria of the gel characterized by the electrochemical equilibrium ($\bar{\mu}^i_\mathrm{gel} = \bar{\mu}^i_\mathrm{res}$) (for $c_\mathrm{s} \neq 0$) and mechanical equilibrium $P_\mathrm{sys} = P_\mathrm{res}$. To determine this zero crossing, we fit the pressure difference $P_\mathrm{res} - P_\mathrm{sys}$ with the phenomenological function $f(L) = a + b/\tan(L - c)$[S1] and interpolate at the zero crossing, i.e., $P_\mathrm{sys} = P_\mathrm{res}$. As a by-product, the bulk modulus G is obtained simply by taking the slope

Table S1: Input parameter sets for the investigated hydrogel architectures

(a) Regular gel

N	α	c_s (M)
20	$\{0.25, 0.5, 1.0\}$	$\{0.0, 0.01, 0.05, 0.1, 0.2, 0.5\}$
25	$\{0.25, 0.5, 1.0\}$	$\{0.0, 0.01, 0.05, 0.1, 0.2, 0.5\}$
30	$\{0.25, 0.5, 1.0\}$	$\{0.0, 0.01, 0.05, 0.1, 0.2, 0.5\}$
37	$\{0.25, 0.5, 1.0\}$	$\{0.0, 0.01, 0.05, 0.1, 0.2, 0.5\}$
45	$\{0.25, 0.5, 1.0\}$	$\{0.0, 0.01, 0.05, 0.1, 0.2, 0.5\}$

(b) Singly/fully-detached chain gel

N	α	c_s (M)	n_{DC}
20	1.0	$\{0.0, 0.01, 0.1\}$	$\{1, 2, 3, 4\}$
25	1.0	$\{0.0, 0.01, 0.1\}$	$\{1, 2, 3, 4\}$
30	1.0	$\{0.0, 0.01, 0.1\}$	$\{1, 2, 3, 4\}$
37	1.0	$\{0.0, 0.01, 0.1\}$	$\{1, 2, 3, 4\}$

(c) Bottlebrush gel

N	α	c_s (M)	m	n
20	1.0	0.0	4	6
25	1.0	0.0	4	6
30	1.0	$\{0.0, 0.01, 0.1\}$	$\{2, 4, 6, 8, 10, 12\}$	$\{4, 6\}$
37	1.0	0.0	4	6
45	1.0	0.0	4	6

(d) Floating-chain gel

N	α	c_s (M)	N_f
20	1.0	0.0	32
25	1.0	0.0	32
30	1.0	$\{0.0, 0.01, 0.1\}$	$\{2, 4, 6, 8, 16, 32\}$
37	1.0	0.0	32
45	1.0	0.0	32

of the P-V curve near the zero crossing:

$$G = -V_{\text{eq}} \frac{\partial P}{\partial V}\bigg|_{\text{eq}} . \tag{1}$$

The error in the bulk modulus is calculated according to the propagation of the error in the volume V and the slope $\frac{\partial P}{\partial V}$:

$$\Delta G = \left(\frac{\partial P}{\partial V}\right)_{\text{eq}} \Delta V_{\text{eq}} + V_{\text{eq}} \Delta \left(\frac{\partial P}{\partial V}\right)_{\text{eq}} \tag{2}$$

The error margins ΔV_{eq} and $\Delta \left(\frac{\partial P}{\partial V}\right)_{\text{eq}}$ are determined using the error bars of the pressure next to the equilibrium point.

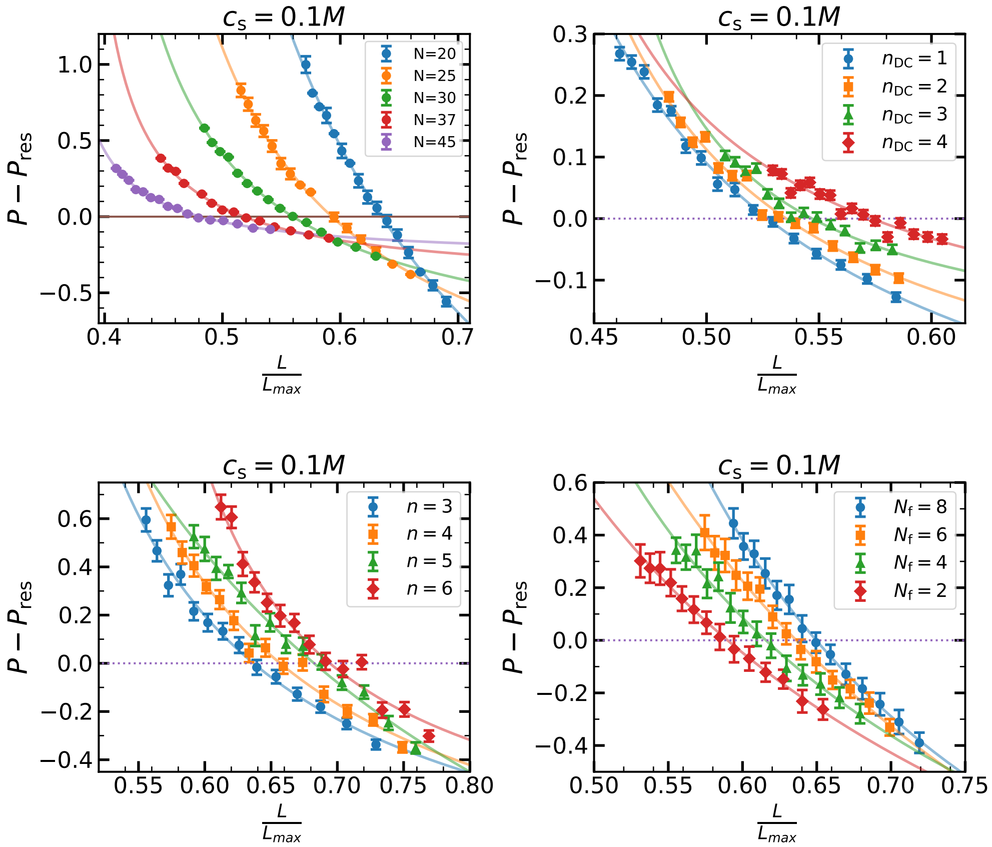

Figure S1: Data points are P-V curves, where osmotic pressure of the system $\Pi = P - P_{\rm res}$ is obtained by changing the concentration of the (a) regular gels, (b) singly-detached chain gels, (c) bottlebrush gels, and (d) floating chain gels. The pressure values on the y-axis are in kPa, and all the systems are coupled to a reservoir of salt concentration 0.1M. The data points are fitted and interpolated with the function $f(L) = a + b/\tan(L - c)$.

2 Benchmarking Scaling Prediction

Simulations of regular hydrogels with full ionization ($\alpha = 1.0$) show a general agreement with the scaling predictions (Figure S2a). However, we observe a non-monotonic dependence of the bulk modulus G on the reservoir salt concentration c_s for a given N. To illustrate this, Figure S2b presents G versus Q_V for $N = 45$ at $\alpha = 0.25$ and 1.0, highlighting that non-monotonicity arises only in the fully charged case. This is further confirmed in Figure S2c, where no non-monotonicity is seen for $\alpha = 0.25$ in different N. In contrast, Figure S2d shows that for $\alpha = 1.0$ the modulus decreases at low c_s and increases again at higher c_s, a trend opposite to experiments where G usually increases monotonically with salt concentration.[S2,S3] Furthermore, the minima of $G(c_\mathrm{s})$ shift to higher values of c_s as the length of the network chain N decreases.

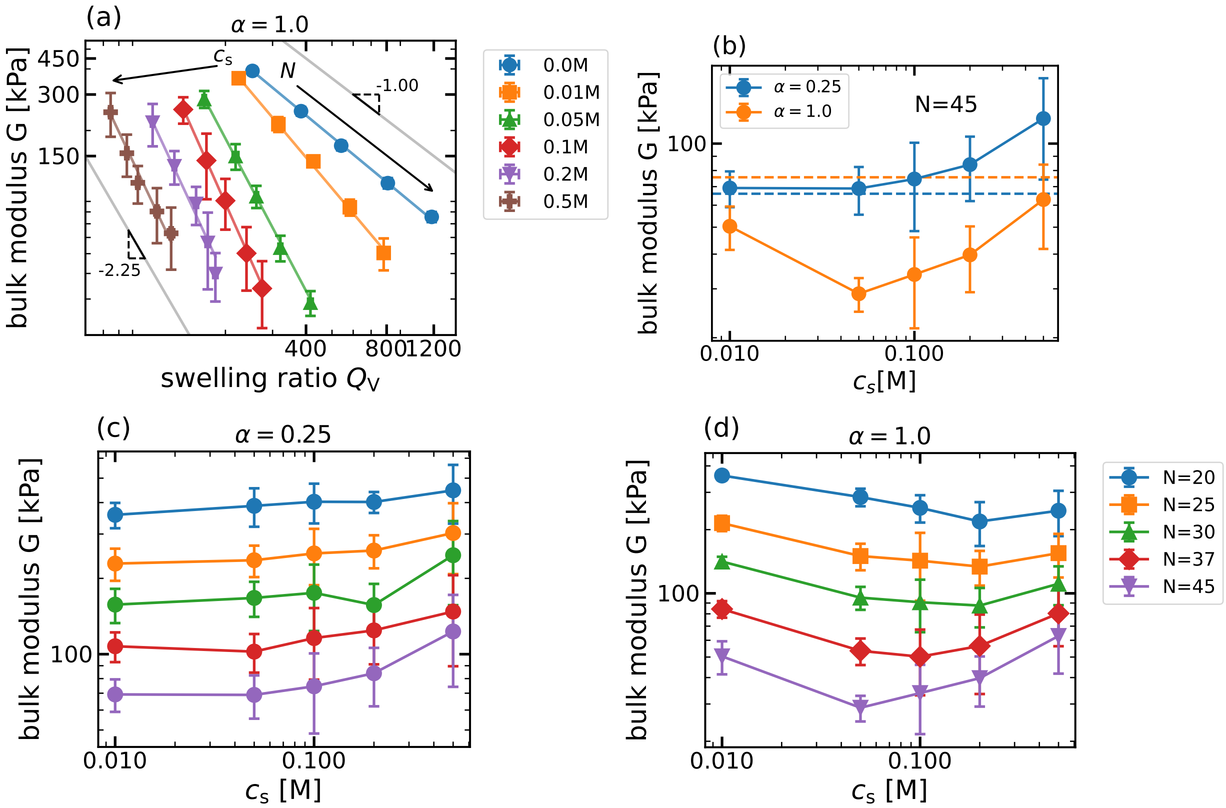

Figure S2: (a): Bulk modulus, G, versus the swelling ratio, Q_V, at various salt concentrations c_s and backbone chain lengths N. The grey lines with slopes of -1 and -2.25 represent the scaling predictions for low and high salt conditions. For each c_s, gels with $N = 20, 25, 30, 37,$ and 45 are considered. Solid lines of each of the data sets at a fixed c_s correspond to error-weighted linear regressions. (b): Bulk modulus, G, versus reservoir salt concentration, c_s for various charge fractions $\alpha = 0.25, 1.0$. The dashed lines corresponds to bulk modulus value at $c_s = 0$M. (c): Bulk modulus, G, versus reservoir salt concentration, c_s for a backbone chain lengths of $N = 20, 25, 30, 37, 45$ and degrees of ionization $\alpha = 0.25$. (d): Bulk modulus, G, versus reservoir salt concentration, c_s, for a backbone chain lengths of $N = 20, 25, 30, 37, 45$ and degrees of ionization $\alpha = 1.0$.

3 Counterion Condensation in Singly and Fully-detached-Chain gel Networks

Firstly, for a clear comparison of the mechanical properties of fully and singly-detached chain gels with reference regular gels, we plot G versus Q_V for chain lengths $N = 20, 37$ in Figure S3. An increasing number of detached chains $n_\mathrm{DC} = 1, 2, 3, 4$ results in increased swelling capacity and decreased bulk modulus compared to regular gels. In the low salt limit $c_\mathrm{s} = 0.0$M, fully-detached chain gels exhibit a higher swelling ratio and elastic modulus compared to singly-detached chain gels, as indicated by the dotted arrows. However, at $c_\mathrm{s} = 0.1$M, fully-detached chains gels still exhibit a higher swelling ratio, but due to large error bars in the bulk modulus, no clear trend is found.

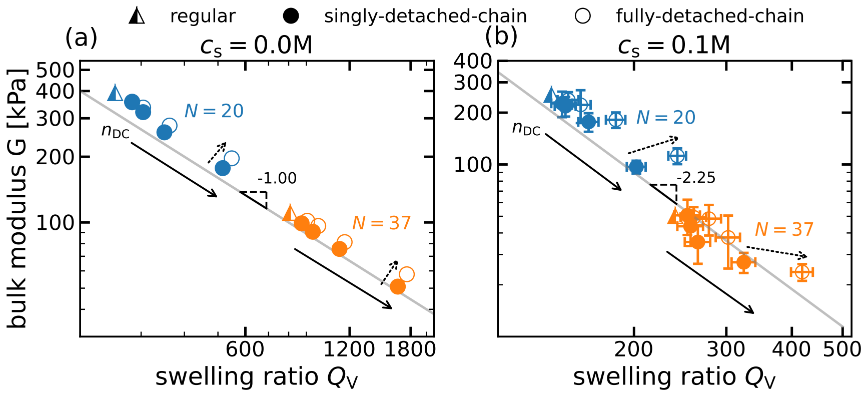

Figure S3: Bulk modulus, G, versus swelling ratio, Q_V, for singly-detachedand fully-detached chain gels at (a) $c_\mathrm{s} = 0.0$M and (b) $c_\mathrm{s} = 0.1$M. Gels with backbone chain lengths $N = 20, 37$ are considered, and the number of detached chains is increased progressively from 1 to 4.

Figure S4 shows the cumulative distribution profile for counterions surrounding a chain N_cond, for severed and unsevered chains for the salt-free case $c_\mathrm{s} = 0$. This cumulative distribution is obtained by counting the counterions within various distances from the monomers of the chain and normalizing it by the number of counterions assigned to the chain. We partition the counterions in the simulation box into chain-specific domains. In a diamond lattice hydrogel, there are 16 chains and we identify each counterion in the simulation box belonging to the unique nearest chain. The left column displays counterion condensation

on the network for singly-detachedchain gels, and the right column shows condensation on the network for fully-detached chain gels. Along the column, the number of severed chains increases $n_{\mathrm{DC}} = 1, 2, 3, 4$. The cumulative counterion distribution of the detached and intact chains is compared to that of chains in the regular gel. The y-axis is normalized by the number of counterions assigned per chain ($N_{\mathrm{total}}/16.0$), so at large distances the profile saturates to the value 1.0. In case of singly-detached chain gels, the distribution profile on the network (intact chains) looks similar to that of a regular gel and remains approximately the same with an increasing number of severed chains, but the distribution profile on the intact chains of the network for fully-detached chain gels decreases with increasing number of severed chains. We see that at any distance $3\sigma < r < 8\sigma$ shows clear differences for the condensation onto intact chains of the network for fully-detached chain gels, hence the chosen value $R_{\mathrm{c}} = 4\sigma$ to characterize the condensation in Table S2 is representative.

For completeness, we show tables with averaged end-to-end distance R_{e} and effective counterion-condensation per chain N_{cond} values calculated separately for networks with singly and fully-detached chains for both types of chains (severed and intact) for all the values of $n_{\mathrm{DC}} = 1, 2, 3, 4$ explored. Note that the value of cumulative distribution N_{cond} in Table S2 is normalized by the respective number of chain types (singly-detached, fully-detached or intact) and R_{e} is normalized by the chain contour length, i.e. 20σ. The analysis confirms that counterion condensation on intact chains of the network for fully-detached chain gels is lower than for the network with singly-detached chain gels, and the differences increase with n_{DC}. The R_{e} values for intact chains of the network for fully-detached chain gels are generally higher than its counterpart network and the difference increases with n_{DC}.

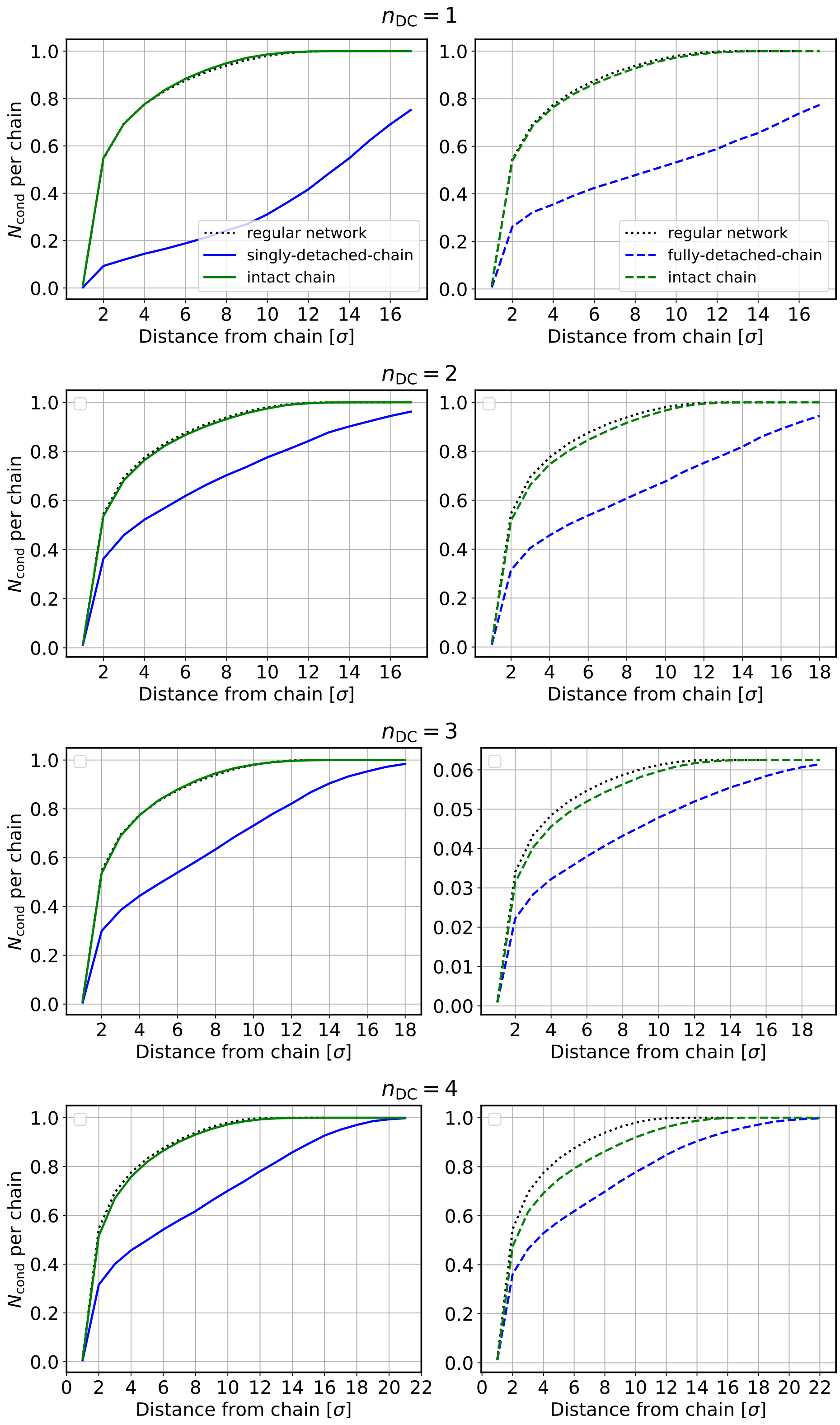

Figure S4: A comparison of cumulative counterion distribution profile around backbone chains of the regular gel with network's intact and singly-detachedchains in the left column and with network's intact and fully-detached chains in the right column. Data is shown for increasing number of detached chains $n_{DC} = 1, 2, 3, 4$

Table S2: Cumulative couterion distribution profile per chain, N_{cond}, and averaged end-to-end distance, R_{e}, for detached and intact chains in gels for all the values of $n_{\mathrm{DC}} = 1 - 4$ explored.

$n_{\mathrm{DC}} = 1$		N_{cond}	R_{e}
Regular		0.0483	0.744 ± 0.002
Singly	detached	0.0090	0.598 ± 0.004
	intact	0.0484	0.738 ± 0.002
Fully	detached	0.0222	0.549 ± 0.003
	intact	0.0477	0.739 ± 0.002

$n_{\mathrm{DC}} = 2$		N_{cond}	R_{e}
Regular		0.0483	0.744 ± 0.002
Singly	detached	0.0326	0.599 ± 0.003
	intact	0.0477	0.759 ± 0.002
Fully	detached	0.0284	0.54 ± 0.002
	intact	0.0466	0.766 ± 0.002

$n_{\mathrm{DC}} = 3$		N_{cond}	R_{e}
Regular		0.0483	0.744 ± 0.002
Singly	detached	0.0277	0.61 ± 0.003
	intact	0.0483	0.758 ± 0.002
Fully	detached	0.0322	0.546 ± 0.002
	intact	0.0456	0.767 ± 0.002

$n_{\mathrm{DC}} = 4$		N_{cond}	R_{e}
Regular		0.0483	0.744 ± 0.002
Singly	detached	0.0285	0.626 ± 0.003
	intact	0.0473	0.775 ± 0.002
Fully	detached	0.0327	0.558 ± 0.002
	intact	0.0433	0.787 ± 0.002

4 Floating-chain gels

To study the effect of counterion delocalization on the mechanical properties of hydrogels, we consider the case of a regular gel co-existing with floating chains. To make a choice for the length of the floating chains, we choose two different lengths $n = N$ and $n = N/2$ for a regular gel with $N = 30$ in Figure S5. In both cases, a similar deviation is observed from the scaling prediction. Hence, we chose the length of floating chains to be the same as the length of the network chain $n = N$.

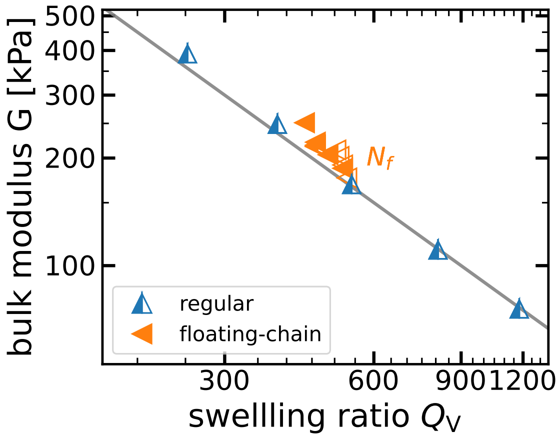

Figure S5: Bulk modulus, G, versus swelling ratio Q_V for floating-chain gel network with length of floating chains half the degree of polymerization of the backbone network chain length $n = N/2$ (shown in empty orange markers) compared to floating chains with $n = N$ (filled orange markers) for the network chain length $N = 30$.

5 Counterion Condensation for Bottlebrush Gels and Floating-chain gels

In Figure 7 in the main text, we observe that floating-chain gels exhibit a substantially higher bulk modulus than bottlebrush gels. This is the result of counterion delocalization that floating chains produce. To quantify the degree of delocalization, we plot the cumulative counterion distribution profile for these two architectures and compare it with the reference regular gel in Figure S6. A higher bulk modulus in the floating-chain gels can be attributed to the large availability of free counterions, which leads to higher ionic osmotic pressure. Hence, at equilibrium, the network with floating chains has a higher elastic modulus and equilibrium volume.

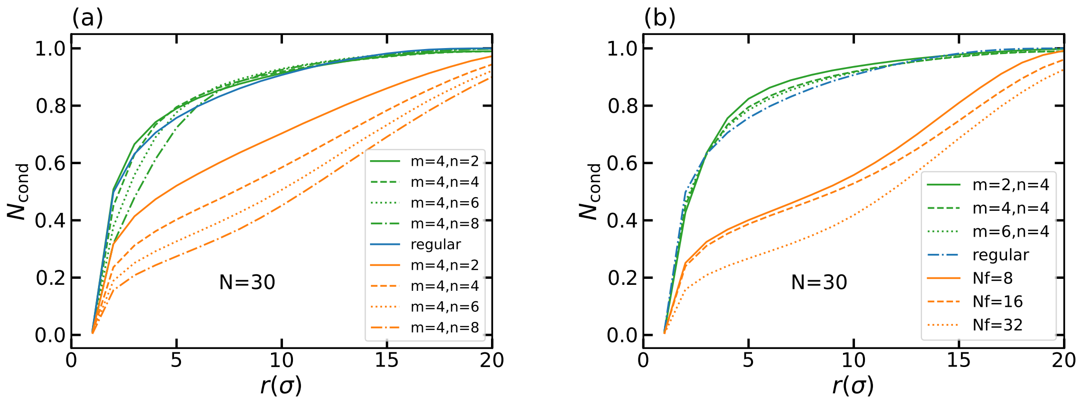

Figure S6: A cumulative counterion distribution profile around regular gels with backbone chain length $N = 30$ compared with bottlebrush gels and floating-chain gels of various architectural parameters (m,n,N_f)

6 Mechanical Properties in the Presence of Salt

In Figure S7, we examine the mechanical properties of all network architectures at a higher reservoir salt concentration $c_s = 0.1$M. Figure S7a shows G plotted against Q_V, where an error-weighted linear fit produces a scaling exponent $\beta = -2.96 \pm 0.05$. We observe that the differences in Q_V and G of the gel with changes in architectural parameters decrease further compared to Figure 8 of the main text for $c_s = 0.01$M. We note that floating-chain gels are particularly affected: despite increasing the number of floating chains $N_f = 1, 2, \ldots, 8$, neither G nor Q_V exhibits a systematic trend and the data cluster around the value of the reference regular gel with only a slight upward trend in G. However, bottlebrush gels with parameters $n = 3, 4, 5, 6$ and $m = 3$ show reduced swelling ratios while their bulk modulus remains nearly unchanged within the error bars compared to the regular gel. This stronger effect at a higher salt concentration on floating-chain gels arises because, at a lower salt concentration, they benefit from counterion delocalization that enhances both modulus and swelling, a mechanism that is suppressed in the presence of additional salt ions from the reservoir. In addition, Figure S7b shows G plotted against αC_p at $c_s = 0.1$ M, where the fit $G \propto C_p^{2.96}$ captures the trend observed in singly-detached-chain gels and regular gels. This slope matches that obtained in Figure S7a but with a positive sign, which is consistent with

the relation $Q_{\mathrm{V}} \propto 1/\alpha C_{\mathrm{p}}$. We confirm this scaling in Figure S8, where plotting Q_{V} versus αC_{p} for all explored architectures shows that the data collapse onto the fit $Q_{\mathrm{V}} \propto 1/\alpha C_{\mathrm{p}}$ for both $c_{\mathrm{s}} = 0.01$ M and $c_{\mathrm{s}} = 0.1$M.

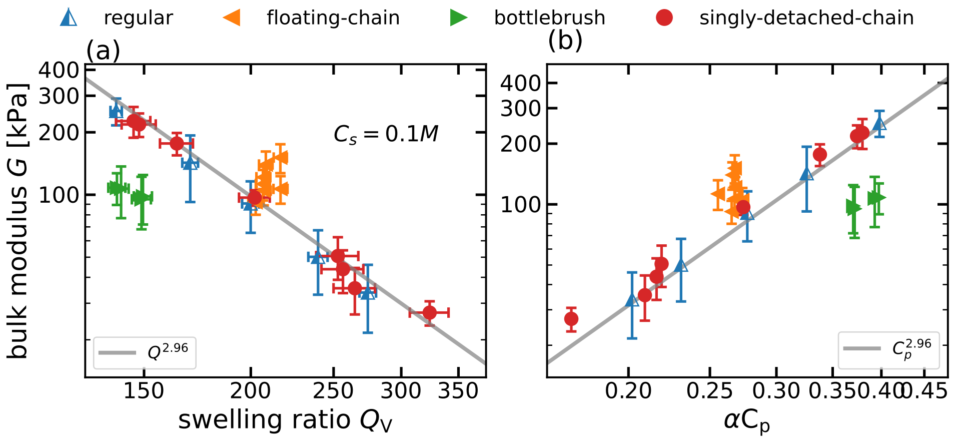

Figure S7: (a): Bulk modulus, G, versus the swelling ratio, Q_V, of networks of various architectures at salt concentrations $c_s = 0.1M$. The grey line with slopes of -2.96 represent the error weighted linear regression fit to the bulk modulus values of regular gels. The network chain length for regular gels are $N = 20, 25, 30, 37$, and 45. The floating chains and bottlebrushes are added to gel with $N = 30$. The number of detached chains $n_{\mathrm{DC}} = 1, 2, 3, 4$ are considered for gels with two backbone chain lengths $N = 20, 37$. (b): Bulk modulus, G, versus equilibrium polymer concentration, αC_{p}, for the same system considered in panel a.

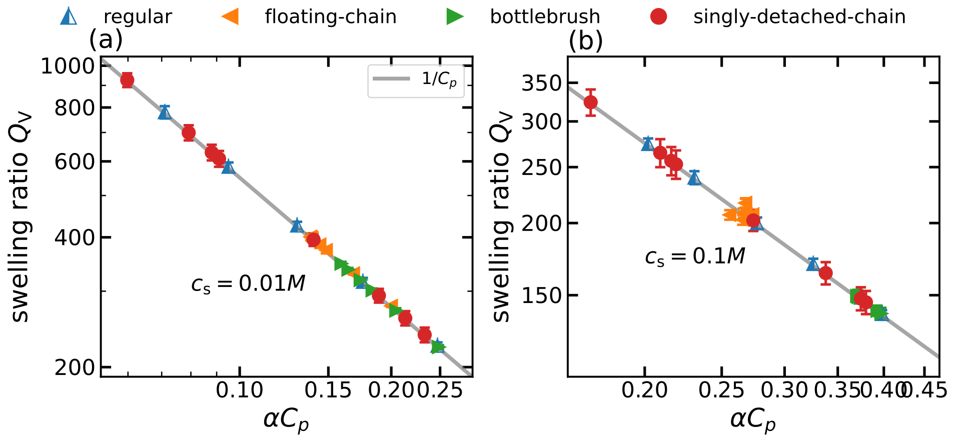

Figure S8: Swelling ratio Q_V versus equilibrium polymer concentration αC_{p} for all the considered network architectures at two reservoir salt concentration, $c_{\mathrm{s}} = 0.01, 0.1$M. All the values follow the relation $Q_V \sim 1/C_{\mathrm{p}}$.

7 Donnan Theory

Donnan theory allows us to describe the partitioning of salt between two phases in the presence of impermeable charges[S1]. In the context of polyelectrolyte hydrogels, the Donnan theory describes the unequal partitioning of salt between an aqueous solution, referred to as the "reservoir", and the gel. For macroscopic gels, an electroneutrality constraint holds, which can be expressed as

$$\alpha c_{\mathrm{p}} + c_{-,\mathrm{gel}} = c_{+,\mathrm{gel}}. \tag{3}$$

Here, αc_{p} is the concentration of impermeable charges (polymer) in the gel, and $c_{-,\mathrm{gel}}$, $c_{+,\mathrm{gel}}$ are the charge concentrations of anions and cations within the gel, respectively. Note that we assumed a purely anionic hydrogel. Because small ions can be exchanged between the gel and the reservoir, an electrochemical equilibrium emerges between these two phases. Mathematically, this equilibrium is described by the equations

$$\mu_-^{\ominus} + k_{\mathrm{B}}T \log\left(\frac{c_{-,\mathrm{gel}}}{c^{\ominus}}\right) + \mu^{\mathrm{ex}}_{-,\mathrm{gel}} - e\psi^{\mathrm{Don}} = \mu_-^{\ominus} + k_{\mathrm{B}}T \log\left(\frac{c_{-,\mathrm{res}}}{c^{\ominus}}\right) + \mu^{\mathrm{ex}}_{-,\mathrm{res}} \tag{4}$$

$$\mu_+^{\ominus} + k_{\mathrm{B}}T \log\left(\frac{c_{+,\mathrm{gel}}}{c^{\ominus}}\right) + \mu^{\mathrm{ex}}_{+,\mathrm{gel}} + e\psi^{\mathrm{Don}} = \mu_+^{\ominus} + k_{\mathrm{B}}T \log\left(\frac{c_{+,\mathrm{res}}}{c^{\ominus}}\right) + \mu^{\mathrm{ex}}_{+,\mathrm{res}}, \tag{5}$$

which express the equality of the electrochemical potentials in the gel and the reservoir. In these equations, $c^{\ominus}$ is an arbitrarily chosen reference concentration, $\mu_i^{\ominus}$ is the value of the chemical potential in the reference state, $\mu^{\mathrm{ex}}_{i,\mathrm{gel}}$, $\mu^{\mathrm{ex}}_{i,\mathrm{res}}$ are the excess chemical potentials in the gel and reservoir and ψ^{Don} is the Donnan potential, which is unknown at this point. Adding the two equations eliminates the Donnan potential and, after some algebra, one obtains the relation

$$c_{+,\mathrm{gel}} c_{-,\mathrm{gel}} = c_{+,\mathrm{res}} c_{-,\mathrm{res}} \cdot \exp\left(\beta \Delta\mu^{\mathrm{ex}}\right). \tag{6}$$

Here, we introduce the abbreviation $\Delta\mu^{\mathrm{ex}} \equiv \mu^{\mathrm{ex}}_{+,\mathrm{res}} + \mu^{\mathrm{ex}}_{-,\mathrm{res}} - \mu^{\mathrm{ex}}_{+,\mathrm{gel}} - \mu^{\mathrm{ex}}_{-,\mathrm{gel}}$ for the difference in the excess chemical potential of an ion pair between the reservoir and the gel. The partitioning of salt ions is typically quantified using a partition coefficient ξ. For an anionic gel, the partition coefficient of salt ion pairs is equivalent to the partition coefficient of anions, given by

$$\xi = \xi_- = \frac{c_{-,\mathrm{gel}}}{c_{-,\mathrm{res}}}. \tag{7}$$

Using Eq. 6, the partition coefficient of the cations can be expressed as

$$\xi_+ = \frac{\exp\left(\beta\Delta\mu^{\mathrm{ex}}\right)}{\xi_-}. \tag{8}$$

Combining Eq. 3 with Eq. 8, we obtain the relation

$$\xi = -\frac{\alpha c_{\mathrm{p}}}{2c_{\mathrm{s}}} + \sqrt{\left(\frac{\alpha c_{\mathrm{p}}}{2c_{\mathrm{s}}}\right)^2 + \exp\left(\beta\Delta\mu^{\mathrm{ex}}\right)}. \tag{9}$$

8 Salt Partitioning

Figure S9 shows the salt partition achieved by hydrogels of various architectures with a bulk salt concentration $c_s = 0.1$M. For a hydrogel with $N = 30$, the number of floating chains is varied between $N_f = 1 - 8$. For the bottlebrush gel, the brush spacing is fixed to $m = 3$ and the brush length is varied $n = 3, 4, 5, 6$. We make a similar observation to Figure 8 of the main text, i.e a regular gel with varying crosslinker density and singly-detachedchain gels follow a same trend, while the bottlebrush gel and floating-chain gels deviate from this trend. Since in this case a maximum of $N_f = 8$ chains are added compared to $N_f = 32$ in Figure 8 of the main text and due to a 10 times higher reservoir salt concentration, no strong deviations are observed for the floating-chain gel network.

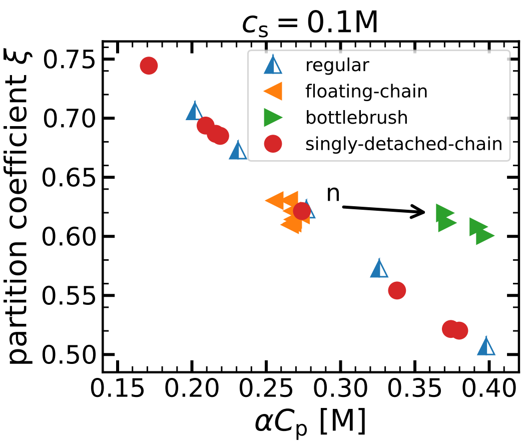

Figure S9: Partition coefficient, ξ, of monovalent salt versus the polymer charge concentration, αc_{p}, for different network architectures. The number of floating chains $N_{\mathrm{f}} = 1, 2, \ldots, 8$ are considered for floating-chain gels.

References

(S1) Landsgesell, J.; Hebbeker, P.; Rud, O.; Lunkad, R.; Košovan, P.; Holm, C. Grand-Reaction Method for Simulations of Ionization Equilibria Coupled to Ion Partitioning. *Macromolecules* **2020**, *53*, 3007–3020, DOI: 10.1021/acs.macromol.0c00260.

(S2) Nisato, G.; Schosseler, F.; Candau, S. Swelling Equilibrium Properties of Partially Charged Gels: The Effect of Salt on the Shear Modulus. *Polymer Gels and Networks* **1996**, *4*, 481–498, DOI: 10.1016/S0966-7822(96)00024-X.

(S3) Skouri, R.; Schosseler, F.; Munch, JP.; Candau, SJ. Swelling and Elastic Properties of Polyelectrolyte Gels. *macromolecules* **1995**, *28*, 197–210, DOI: 10.1021/ma00105a026.
